# Comprehensive Peak Characterization (CPC) in Untargeted LC–MS Analysis

**DOI:** 10.3390/metabo12020137

**Published:** 2022-02-02

**Authors:** Kristian Pirttilä, David Balgoma, Johannes Rainer, Curt Pettersson, Mikael Hedeland, Carl Brunius

**Affiliations:** 1 Department of Medicinal Chemistry, Uppsala University, SE-75123 Uppsala, Sweden; david.balgoma@ilk.uu.se (D.B.); curt.pettersson@ilk.uu.se (C.P.); mikael.hedeland@ilk.uu.se (M.H.); 2 Institute for Biomedicine, Eurac Research, Affiliated Institute of the University of Lübeck, 39100 Bolzano, Italy; johannes.rainer@eurac.edu; 3 Department of Biology and Biological Engineering, Chalmers University of Technology, SE-41296 Gothenburg, Sweden; carl.brunius@chalmers.se; 4 Chalmers Mass Spectrometry Infrastructure, Chalmers University of Technology, SE-41296 Gothenburg, Sweden

**Keywords:** metabolomics, untargeted, peak characterization, peak detection, XCMS, false peaks, peak filtering, data processing, algorithm, data quality

## Abstract

LC–MS-based untargeted metabolomics is heavily dependent on algorithms for automated peak detection and data preprocessing due to the complexity and size of the raw data generated. These algorithms are generally designed to be as inclusive as possible in order to minimize the number of missed peaks. This is known to result in an abundance of false positive peaks that further complicate downstream data processing and analysis. As a consequence, considerable effort is spent identifying features of interest that might represent peak detection artifacts. Here, we present the CPC algorithm, which allows automated characterization of detected peaks with subsequent filtering of low quality peaks using quality criteria familiar to analytical chemists. We provide a thorough description of the methods in addition to applying the algorithms to authentic metabolomics data. In the example presented, the algorithm removed about 35% of the peaks detected by XCMS, a majority of which exhibited a low signal-to-noise ratio. The algorithm is made available as an R-package and can be fully integrated into a standard XCMS workflow.

## 1. Introduction

The aim of untargeted metabolomics is large-scale profiling of the metabolome [1], i.e., all small metabolites (<2000 Da) in biological samples, representing the end-result of all intra- and extracellular processes in addition to exogenous compounds originating from environmental exposures during the life course, e.g., microbiota, diet and medication [2,3]. The field of metabolomics has been growing steadily in the last two decades, primarily due to advances in analytical instrumentation as well as new algorithms suitable for the high dimensional data generated. In targeted analysis, a subset of metabolites believed to be descriptive of the studied phenomena are analyzed, resulting in data of relatively low complexity. Such a selection is generally not made in untargeted metabolomics, being aimed predominantly at hypothesis generation. Consequently, the identity of most analytes is not known *a priori* and considerable effort is spent identifying the detected analytes [4]. In addition to metabolomics, untargeted analysis is also useful in several other research areas, such as emerging pollutants [5,6], doping control [7,8], and exposomics [9].

Liquid chromatography coupled to high resolution mass spectrometry (LC–HRMS) has emerged as a key technique for untargeted metabolomics. Its popularity is due to the high sensitivity of detection in combination with the wide coverage of compounds of different chemical properties. In addition, analyte fragmentation and isotopic patterns can be readily obtained, facilitating identification of the detected compounds. A biological sample, e.g., from plasma, urine or other fluid or tissue, may contain hundreds to thousands of detectable metabolites [1,10,11]. In addition, each of these may be detected as several different adducts and fragments [12]. As a result, manually searching through the immensely complex data for chromatographic peaks is highly impractical, if not impossible. Instead, various algorithmic approaches have been developed to automate this process. Several proprietary and open source software solutions have been developed over the years for this purpose [13]. Two of the most commonly used open source software are XCMS [14] and MZmine [15]. While XCMS is implemented as an R package, and is thus especially suitable for automation and customizable and reproducible high throughput analysis, MZmine is implemented in Java and provides an easy-to-use graphical user interface with emphasis on manual inspection of the data. They utilize highly similar methodologies and, consequently, provide similar, but not identical results [16].

In brief, the feature detection workflow can be divided into a number of discrete steps: (i) extraction of ion traces that are coherent over a sufficiently long time window in which peaks may be present; (ii) performing peak detection on these extracted ion traces; (iii) adjusting retention times over the entire elution range; and (iv) grouping common peaks across samples into features (correspondence). All these steps entail algorithmic decisions and require parametrization, which will have a profound effect on the outcome [17]. The algorithms utilized for peak detection by these software are designed to be very sensitive, in order to minimize false negative peak detection. It has, however, been noted that this high sensitivity may lead to a high abundance of false positive peaks [16,18,19]. These additional peaks add noise to the data with negative effects on both retention alignment and correspondence, and consequently also to downstream data analysis and biological inference. In our experience, several of the discriminant features from data analysis, when inspected, originate from such false positive peaks. Conventional methods to mitigate this problem include using proxy measures to remove features of low quality. One such approach is to reject features with a high relative standard deviation among quality control samples injected throughout the injection sequence (e.g., RSD_QC_ > 30%) [20,21]. However, the quality of chromatographic peaks, in these cases, is assessed solely on their repeatability over an injection sequence, which may in fact not be related to the peak characteristics or the predictive information provided by a feature. Moreover, the signal measured in QC samples might not always represent each study sample completely, hence potentially interesting features might also be removed by such a pre-filtering step.

In conventional analytical chemistry, several analytical measures, such as signal-to-noise ratio (SN) and peak asymmetry, are frequently used to determine peak quality. However, little such information is readily available in conventional peak picking software, such as XCMS, which makes filtering false peaks difficult. However, a number of different strategies for removing false peaks from the dataset have been presented in the past. For instance, Myers et al. published an improved peak detection algorithm based on continuous wavelet transform in 2017 [22], which according to the authors, addresses many of the issues that they previously reported [16]. In terms of filtering peaks with poor quality, there have also been examples of classification models that use peak quality characteristics to train models that can predict whether a peak is trustworthy. As an example, Borgsmüller et al. published a paper describing a workflow that utilizes a Support Vector Machine (SVM) model for this purpose [23]. The developed workflow, called WiPP, exhibited excellent performance for data obtained at high concentration, whereas it struggled at low concentration, detecting 95% and 42% of manually curated peaks, respectively. Similarly, the package ‘MetaClean’ was presented by Chetnik et al. in 2020 [24], which at the time of publication, implemented 24 different classifiers of various types (e.g., SVM and Random Forest). More direct approaches to classify detected peaks, such as that which is presented here, have also been reported in the past. For example, in 2019, Kantz et al. [25] published an approach using image-based deep learning for classification and provided example scripts for processing in R but the code is not provided as an R-package. The developed deep learning algorithm had a success rate of 88% in retaining true peaks and 89% in removing false peaks. Another example was published in the same year by Melnikov et al. [26], which also utilize deep learning neural networks and is made available in the python package ‘peakonly’ but does not have an R implementation. Their algorithm exhibited excellent performance with a precision of close to 97% in detecting true positive peaks. A similar tool available as a python package is ‘NeatMS’ [27], which also utilizes neural networks for peak classification. Finally, in a recent publication, Jirayupat et al. [28] take a somewhat different approach and instead utilize an image-based neural network model for processing two-dimensional maps of LCMS raw files for detection of relevant signals. While these tools exhibit excellent performance with reported True Positive Rate (TPR) and True Negative Rate (TNR) ≥97%, neural network models often require learning from extensive training material and, in addition, require deep knowledge in bioinformatics and machine learning and thus come with a steep learning curve in terms of their implementation. Furthermore, among these tools, only that presented by Kantz et al. [25] has an R implementation, albeit only as an example script. Thus, in light of the popularity of XCMS for data processing, we stipulate that there is a need for tools for characterization of peaks by standard analytical chemistry metrics that can be easily integrated into an R-based, XCMS-centered workflow.

With the aim of improving the overall data quality, we herein present the Comprehensive Peak Characterization (CPC) algorithm, which characterizes and filters the peaks from untargeted MS-based analysis. In the presented manuscript, we first provide a theoretical overview of the CPC algorithm followed by results and observations obtained when applying the algorithm to a real metabolomics dataset. While the algorithms presented herein were originally developed for metabolomics applications, it should be noted that they are of a general nature and have wider applicability to other areas of untargeted LC–MS- or GC–MS-based analysis. The CPC algorithm is made available in the R package CPC that is seamlessly integrated with the XCMS workflow. As the other tools mentioned above do not integrate easily with XCMS, we have opted not to compare them with CPC as a fair benchmarking would require identical data to be processed under ideal conditions. The package will be submitted to Bioconductor and its source code is available on GitHub (https://www.github.com/krispir/cpc/; accessed on 28 January 2022). Documentation and a tutorial is available at https://krispir.github.io/cpc (accessed on 28 January 2022).

## 2. Theory and Methodology

**Figure 1:**
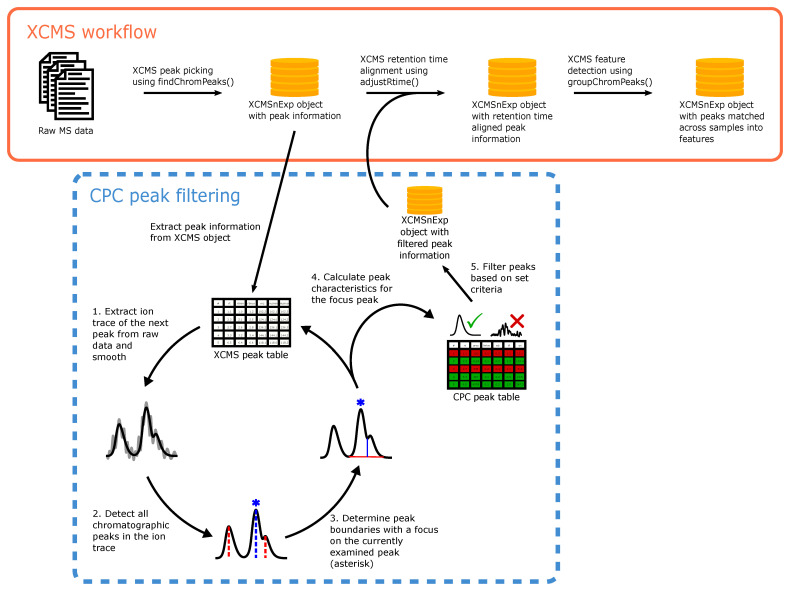
Integration of the CPC algorithm into the XCMS workflow. Following peak detection, the CPC algorithm works directly on the generated *XCMSnExp* object and returns a similar but filtered object that can be used directly in subsequent steps of the XCMS workflow. For each peak in the XCMS peak table, the ion trace is extracted from the raw data and the smoothed trace is calculated together with its second derivative (step 1). All peaks in the ion trace are detected by the CPC algorithm (step 2), while keeping track of the peak reported by XCMS (blue asterisk). Baseline expansion is then performed (step 3), after which peak characteristics are calculated and stored in a table corresponding to the XCMS peak table (step 4). The peak characteristics can then be used to filter the peak table (step 5) before further processing using XCMS.

The peak picking in XCMS provides a list of potential chromatographic peaks, thus allowing for a targeted evaluation of potential peak regions rather than operating on the entire raw data, which would be more computationally expensive. The primary purpose of the CPC algorithm is thus to filter the detected peaks from all files directly after peak detection, which will reduce the data complexity in the subsequent alignment and feature detection steps. This will result in less spurious matching of noise features and thereby improve the quality of the final feature table. An overview of the CPC algorithm and its integration into the XCMS workflow is presented in Figure 1.

**Figure 2:**
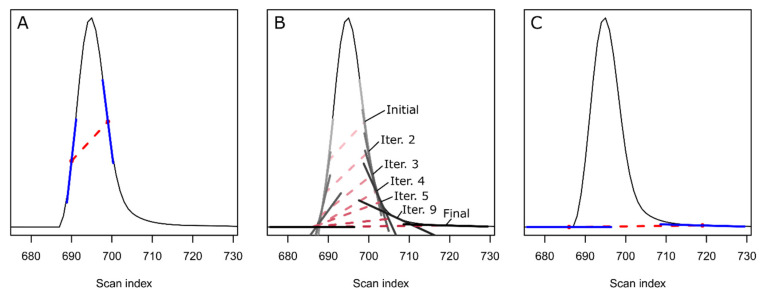
The baseline expansion algorithm initiates at the inflection points of the peak (**A**). The slope difference between the tangents (blue lines) and the current baseline (red dashed line) is calculated. The difference threshold used to terminate the expansion is calculated from the liftoff (% of slope difference between front tangent and initial baseline at inflection points) and touchdown (% of slope difference between tail tangent and initial baseline at inflection points) parameters. The algorithm iteratively expands the baseline boundaries (**B**) until the slope difference is below the threshold on both the front and tail end of the peak (**C**).

For each peak, the CPC algorithm retrieves the extracted ion trace (EIC) from the raw data with an *m/z* window size set by a user-specified ppm parameter (by default the same value as used for XCMS peak picking). The smoothed EIC and its first and second derivatives are then calculated using either a Savitzky–Golay filter (default) or a moving window mean smoother, as specified by the user. Peak apices are detected as local negative minima cradled by inflection points (zero-crossings) along the second derivative. Baseline expansion along the EIC is then performed to locate the boundaries of detected peaks using a modified ApexTrack algorithm [29]. In brief, an initial baseline is set between the front and tail inflection points of the peak (Figure 2A). The baseline bounds are then expanded (Figure 2B) until the slope differences between both the front and tail boundaries and the expanded baseline are below the threshold (Figure 2C). The termination threshold is set using the parameters liftoff and touchdown, given as percent of the initial slope differences (default: liftoff 0% and touchdown 0.5%).

**Figure 3:**
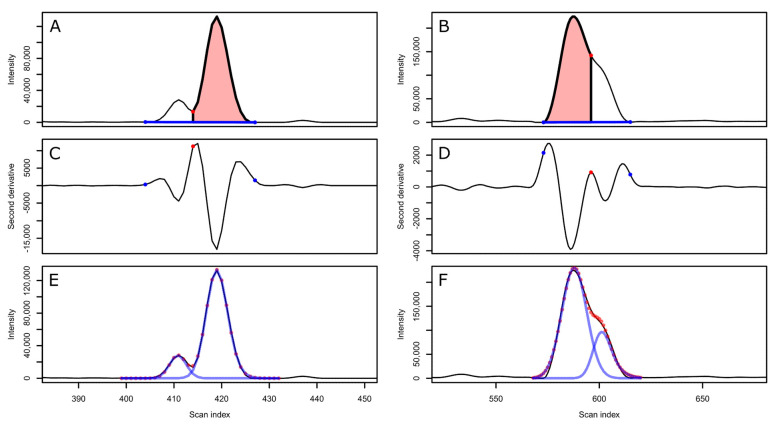
Illustration of the result of CPC processing on extracted ion chromatograms (EIC) representing two peak clusters (**A**,**B**) with their respective second derivatives (**C**,**D**). The peaks currently being processed are indicated in red shading and peak cluster baselines as blue lines (**A**,**B**). For valley boundaries (**A**,**C**), the lowest point along the EIC between the peak apices is set as the boundary. For shoulder boundaries (**B**,**D**), second derivative maxima are instead used. More accurate peak characteristics for the same peak clusters can be obtained through deconvolution (**E**,**F**).

After baseline expansion, co-eluting peak clusters are detected as peaks with overlapping baseline bounds and the baseline bounds for the clusters are updated, taking into account the degree of overlap between peaks, resulting in valley (Figure 3A,C,E), shoulder (Figure 3B,D,F), and rounded (not shown) peak boundaries. The peak cluster boundaries consequently represent approximations, although more precise peak characteristics can be obtained through deconvolution (Figure 3E,F). However, this procedure is computationally expensive and consequently not recommended for the purpose of filtering peaks, where the baseline expansion boundaries provide sufficient accuracy. For deconvolution parametrization, the reader is referred to the documentation of the CPC package.

**Figure 4:**
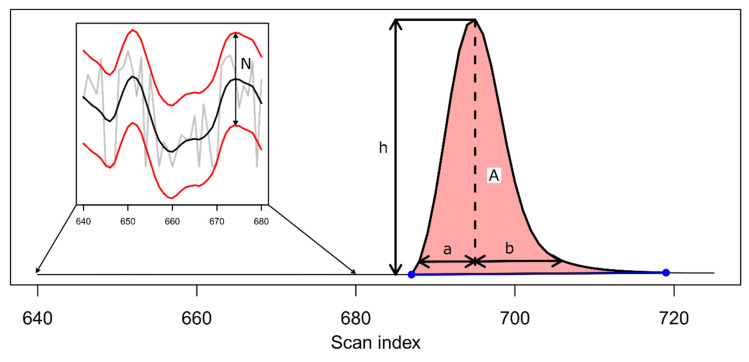
Calculated CPC peak characteristics. Noise (N) is estimated across all non-chromatographic peak scans as the mean peak-to-peak distance between adjacent local maximum/minimum pairs. The peak height is calculated using the determined baseline. The peak area (A) is determined using the trapezoid method between the peak bounds (blue points). The width of the front (a) and tail (b) width is determined at 10% peak height.

Peak quality characteristics (Figure 4) are calculated based on the results from the baseline expansion algorithm. The peak area is calculated between the peak bounds using the trapezoid method. The noise (N, Figure 4) is estimated as the mean absolute peak-to-peak difference for all non-peak scans along the original ion trace. This is used to calculate the signal-to-noise ratio (S/N) for the peak as 2 h/N, where h is the peak height and N is the noise, as recommended by the 2019 European Pharmacopeia (Figure 4) [30]. The base width, width at 5%, and 10% peak height along with full width at half maximum (FWHM) are then calculated using the peak boundaries determined from the baseline expansion. In addition, the front and tail widths (a and b, respectively, Figure 4) are calculated at 10% peak height and used to determine the tailing factor as b/a [31]. The algorithm will return all calculated peak characteristics (a characterized peak list can be obtained from the CPC object using the cpt() function). Finally, the CPC algorithm allows filtering of peaks according to several criteria. By default, peaks will be initially filtered if they do not exhibit a characteristic peak signature in the second derivate, i.e., a negative minimum cradled by inflection points. Then, detected peaks with too few data points between the inflection points in the second derivative (default: 3) as well as between the peak bounds (default: 7) can be filtered. In addition, peaks can be filtered based on their S/N (default: 10) as well as the tailing factor (default: not used). All the filtering criteria can be customized by the user.

## 3. Results and Discussion

The algorithm was applied to a dataset from a previously reported study on hydrogen gas attenuation of noise-induced hearing loss in guinea pigs [32]. The full data are available for download via the MetaboLights repository (unique identifier: MTBLS2418). A total of 57 files, including QC sample injections, were processed using XCMS with and without CPC filtering. XCMS detected 1250–5397 peaks per sample with an average of 2287 peaks. The total runtime for the CPC processing was approximately 40 min with a mean processing time per sample of 42.8 s on an HP EliteBook 840 G5 with an Intel Core i7-8550U CPU and 32 GB RAM. In comparison, the XCMS peak picking required on average ca 1.3 min per sample on the same machine. In this example, the CPC algorithm removed approximately 45,000 of the peaks detected by XCMS, corresponding to roughly 35% (Table 1).

**Figure 5:**
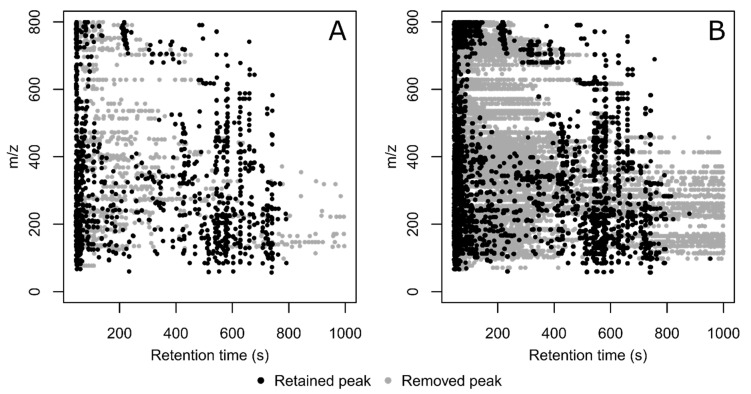
Distribution, in retention time and *m/z* value, of peaks retained (black circles) and removed (gray circles) by CPC. Exemplified by the peaks from the first QC sample injection (**A**, n = 1) and in all collected QC sample injections (**B**, n = 15) of the mouse perilymph experiment.

Despite the large number of removed peaks, only 57 features were in fact removed in the filtered data. However, both the percentage of filled peaks and the percentage of peaks were associated with a feature increase when CPC was applied. This is mainly due to the distribution of removed peaks in the *m/z*—retention time space as is exemplified in Figure 5, which shows removed and retained peaks in the first QC sample injection (Figure 5A) and all QC sample injections (Figure 5B). The primary consequence of this distribution is that most of the removed peaks would not become associated with a feature. However, an important aspect is that without filtering, all these peaks are present during both retention alignment and correspondence and may therefore affect the outcome of those processing steps. Since the peaks (and consequently the features) kept after correspondence in the filtered and non-filtered data, respectively, were similar, the proportion of features passing the RSD_QC_ ≤ 30% filter was highly similar between the two datasets. These results confirm our intuition that, whereas RSD_QC_ provides a good estimate of feature stability in an analytical run, it does not provide a good estimate of peak quality per se. In fact, we would argue that the RSD_QC_ and the CPC approaches are complementary as they target different aspects of the data quality.

**Figure 6:**
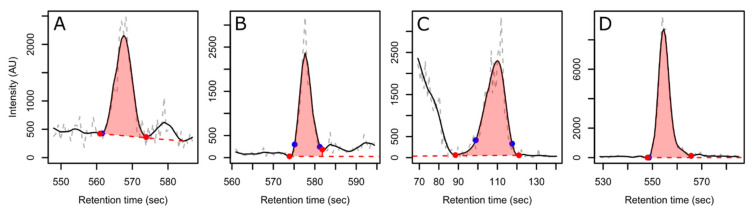
Examples of retained peaks in the dataset selected at random from each quartile of the signal-to-noise ratio (**A**: S/N < 26.13, **B**: 26.13 ≤ S/N < 52.12, **C**: 52.12 ≤ S/N < 117.4, and **D**: 117.4 ≤ S/N). The raw ion trace is shown in dashed grey, the smoothed ion trace is in solid black, the detected baseline is in dashed red, and the detected peak boundaries are the red (CPC) or blue (XCMS) filled circles. For more examples, see Figures S1–S4 in the Supplementary Materials.

We found that the peak expansion algorithm used in CPC provides reasonable estimates of the peak boundaries for both tailing and fronting peaks across a wide range of signal-to-noise ratios (Figure 6).

**Figure 7:**
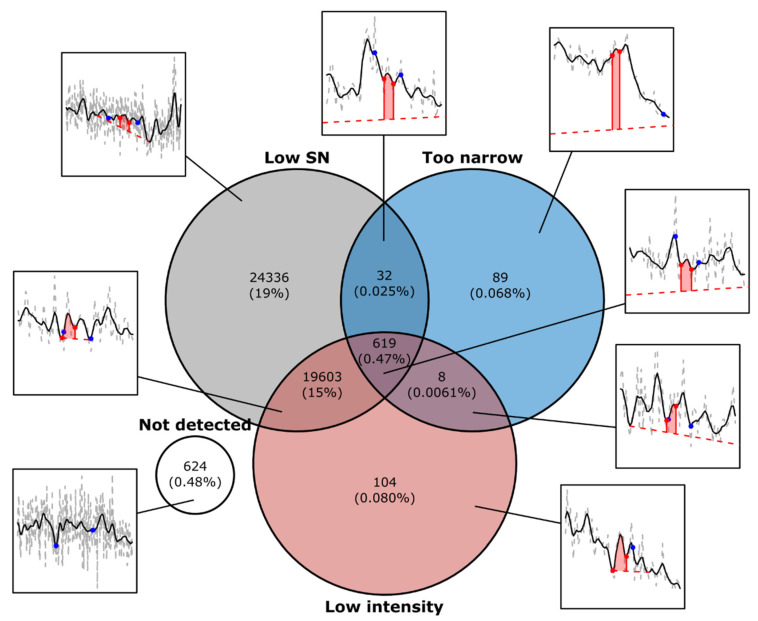
Distribution of removed peaks according to the CPC filters signal-to-noise, peak width, and peak intensity. The satellite boxes show examples of peaks selected from the different subsets. For more examples, see Figures S5–S12 in the Supplementary Materials. The blue dots in the chromatograms indicate the peak bounds reported by XCMS and the red shaded area between the red dots, and the dashed red line indicates the peak region and baseline, respectively, determined by CPC.

In this dataset, the vast majority of removed peaks were filtered due to a low signal-to-noise ratio, which frequently, but not always, coincided with a low total intensity (Figure 7). We observed that a large proportion of the removed peaks originated from periodic fluctuation in the baseline intensity, likely caused by the motion of the LC pump heads (data not shown). Our results are in line with previous research, which has identified a large proportion of false positive XCMS peaks as noise artifacts [16,18,19]. We also observed that the CPC peak filtering appear well suited to detect and remove this type of false peaks.

**Figure 8:**
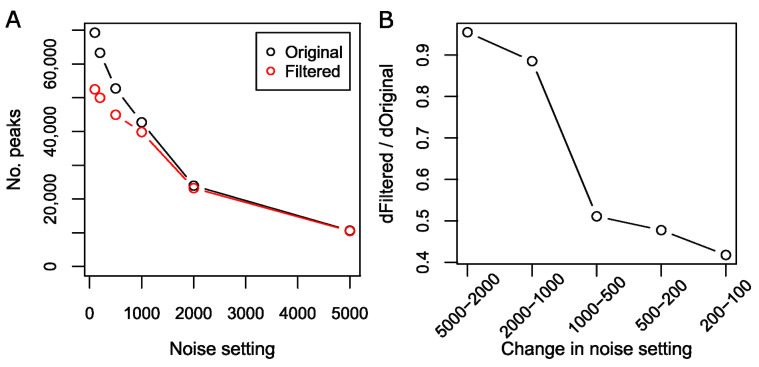
Effect on number of peaks detected vs. retained when changing the noise setting in XCMS peak picking. The number of detected peaks decrease dramatically when the noise setting is increased incrementally from 100 to 5000 (black circles, **A**) and the number of these that are retained by the CPC algorithm appears to converge with the number of detected peaks at approximately 2000 (red circles, **A**). This can also be seen in the ratio between the increase in retained peaks (dFiltered, **B**) and increase in detected peaks (dOriginal, **B**). As the noise setting is incrementally decreased from 5000 to 100, the proportion of removed peaks becomes successively higher.

The XCMS noise threshold parameter strongly influences the peak detection and due care must be taken by the user in setting the peak picking parameters to avoid detecting low-intensity noise as peaks. When applying the CPC algorithm to XCMS results obtained from successively lowering noise thresholds (Figure 8), i.e., allowing the detection of smaller peaks, we observed a successive increase in the proportion of peaks removed by CPC. In addition to highlighting the association between instrument noise and peak artifacts, the results demonstrate the ability of CPC to remove such noise artefacts.

Although the proportion of removed peaks increases with lower noise thresholds, not all additional peaks at the lower thresholds are removed by CPC and may therefore reflect actual analytes at detectable, albeit low intensities. This indicates that optimization of the noise threshold parameter in XCMS is insufficient to properly manage exclusion of false negative peaks. Instead, a combination of tuning the noise threshold and applying algorithms, such as CPC to filter the resulting peak list, allows the inclusion of as many informative peaks as possible while maintaining a minimum of false peaks. Additionally, our results suggest that even with thorough parameter optimization, XCMS may be overly optimistic in estimating S/N as most of the filtered peaks exhibit an S/N that is too low.

As mentioned, the presence of these false peaks in the data during correspondence may have an effect on the grouping of peaks into features. For that reason, in a similar experiment, the ‘bw’ parameter was varied in the correspondence step of the XCMS workflow. This parameter defines the bandwidth of the density function and thereby relates to how much the retention times of peaks (between samples) are allowed to vary within a feature. Thus, with an increased number of randomly distributed peaks, the chance for spurious matches of peaks into features increases. For UHPLC-derived data with minimal retention time variation, a suitable setting for this parameter is normally around 1–2. However, with a larger drift in retention times, e.g., from sub-optimally prepared samples, it may become necessary to increase this parameter. For this reason, bw settings of 1, 2, 5, and 10 were investigated. We observed a clear effect of the CPC processing in relation to the bw parameter: As bw is increased, the number of peaks that are associated with a feature after correspondence increases in both the unfiltered and filtered datasets. However, the increase is much greater in the unfiltered data (Supplementary Materials, Figure S13). We also observed that there were fewer filled peaks for the filtered data, especially at higher bw (Supplementary Materials, Figure S14). We interpret this to mean that there are less spuriously matched features in this dataset that require peak filling. In addition, as a side-effect of peaks that lie near to each other in the *m/z*—retention time space, we have observed that XCMS occasionally associate more peaks to features than there are samples. This effect was somewhat mitigated by CPC (Supplementary Materials, Figure S15). Finally, we conducted a deeper investigation of the features that were associated with peaks that CPC removed. The majority of the features were either largely intact (i.e., the remaining peaks from the other samples were still contained in the same feature) or completely removed (i.e., most or all peaks within feature were filtered), representing either high or low quality features (Supplementary Materials, Table S1). As bw increased, not only did the number of such high and low quality features increase, but also the occurrence of features that were partly intact (Supplementary Materials, Table S1). This would indicate first that false peaks contribute to erroneous correspondence, especially at higher bw settings. Second, it suggests that by removing these peaks, CPC filtering should help in mitigating this problem.

To assess the performance of the algorithm to determine whether a peak reported by XCMS should be kept or filtered, we manually curated a subset of peaks and compared the outcome with that reported by CPC. To this end, 144 peaks were selected by random sampling of 36 peaks from each quartile of XCMS reported peak intensities. Each peak was then subjected to an expert assessment based on the general peak shape, approximate signal-to-noise ratio, and the width of the peak and then contrasted with the CPC algorithm (Supplementary Materials, Table S2). The CPC algorithm exhibited an overall True Positive Rate (TPR), True Negative Rate (TNR), and F_1_ score of 90.8%, 87.7%, and 91.3%, respectively. While these values are somewhat modest in relation to those reported for the best performing deep learning algorithms, they compare favorably to the other approaches in reducing false peaks.

**Figure 9:**
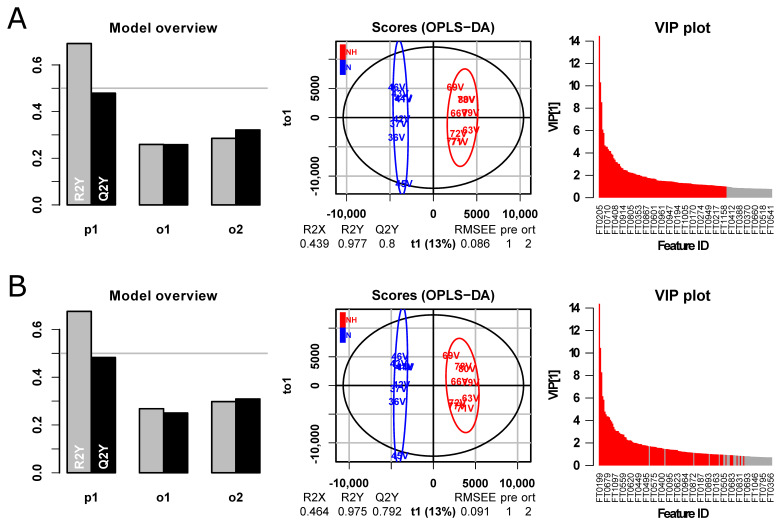
OPLS-DA models fitted on the guinea pig perilymph metabolome from study groups Noise vs. Noise + H_2_ [32] without (**A**) and with (**B**) CPC filtering. As the models show equal discriminatory ability, the CPC filtering does not appear to have removed features with biologically important information. Features with VIP ≥ 1 in the models fitted on data without CPC filtering (**A**) were matched to the features in the filtered data and are colored red in the VIP plots of both models.

While the purpose of the peak filtering is to remove noise artefacts and other false peaks that exhibit spurious associations with the research question, consequently leading to less time spent identifying false positives, it should not negatively impact biological inference. As an example of the potential effects of CPC filtering on inference, we performed an OPLS-DA on the effects of Noise vs. Noise + H_2_ on the guinea pig perilymph metabolome, which was the primary focus in the original study [32]. The data analysis was performed on both the original (Figure 9A) and filtered (Figure 9B) datasets. Some change in the order of the features of interest (VIP ≥ 1) was observed between the models (colored red in VIP plots in Figure 9). However, models fitted using the original and filtered datasets exhibited similar discriminatory power (Q2, Figure 9), indicating that the CPC filtering did not impact biological inference.

## 4. Materials and Methods

### 4.1. Software

All development of the CPC algorithm and package was carried out using the R and C++ language program and any data analysis was performed in the R statistical language environment (version 4.1.1). The package was built using devtools (version 2.4.2) and usethis (version 2.1.2) R packages in RStudio (version 1.3.1093, RStudio, Boston, MA, USA). Raw data files were converted from. RAW to. CDF files using Databridge (version 3.5, Micromass UK Ltd. Manchester, England). Further data preprocessing (i.e., peak picking, retention alignment, correspondence, and peak filling) was performed using XCMS (version 3.14.1) [14]. Orthogonal Projection to Latent Structures Discriminant Analysis (OPLS-DA) models were fitted using the R-package ropls (version 1.24.0).

### 4.2. Untargeted Metabolomics Analysis of Guinea Pig Perilymph Samples

The sample preparation protocol and analytical method used to analyze the guinea pig perilymph samples have been reported in detail elsewhere [32]. In short, LC–MS data were acquired from perilymph samples obtained from 42 guinea pigs along with 15 quality control (QC) sample injections. The individuals were divided into four different study groups that were either exposed to noise with subsequent hydrogen gas administration (*Noise+H*_2_, n = 17) and without (*Noise*, n = 15), or control groups that were not exposed to noise with hydrogen gas administration (*H*_2_, n = 7) and without (*Control*, n = 3). Protein precipitation was achieved by addition of 20.0 µL cold acetonitrile to 5.0 µL sample aliquots. After centrifugation (21,000× *g*, 4 °C, 15 min), the samples were analyzed without further treatment. LC–MS analysis was performed using a Waters ACQUITY I-class UPLC system (Waters Corp., Milford, CO, USA) coupled to a Synapt G2-S QTOF High Resolution Mass Spectrometer (Waters). Chromatographic separation was achieved in HILIC mode with a Waters BEH Amide column (50 × 2.1 mm i.d., 1.7 µm particle size, 100 Å pore size) fitted with a Waters VanGuard BEH Amide (5 × 2.1 mm i.d., 1.7 µm particle size, 100 Å pore size) guard column. See supplementary text or reference [32] for further details.

### 4.3. Data Processing with XCMS and CPC

Only mass peaks between 45 and 1000 s were processed by XCMS. Parameter selection for XCMS was performed using the proposed workflow presented in the XCMS vignette in combination with our experience in working with these algorithms. Peak picking was performed using the centWave algorithm with parameters ppm = 50, peakwidth = c(5, 40), snthresh = 10, fitgauss = TRUE, noise = 200, integrate = 2, prefilter = c(5, 1000), and verboseColumns = TRUE, mzdiff = 0.01. Retention alignment using the obiwarp method with parameters binSize = 0.01, centerSample = 33 (middle QC sample), response = 1, gapInit = 2.5, and gapExtend = 4.0. Correspondence was performed using the ‘peak density’ algorithm with parameters minFraction = 0.8, binSize = 0.02, and bw = 2. Peak filling parameters as well as all other parameters in the other functions were kept as default. CPC processing parameters, selected based on observations during the development of the algorithm, were ppm = 50 (*m/z* window used when extracting raw ion traces), min_pts = 7 (minimum points between peak bounds), min_inf_width = 3 (minimum points between inflection points in the second derivative), min_sn = 10 (minimum signal-to-noise ratio), min_intensity = 2000 (minimum peak area), min_shoulder_pts = 3 (minimum number of points between the bounding second derivative maxima and the peak apex of shoulder peak pairs), min_rounded_pts = 3 (minimum number of points between the bounding second derivative maxima and the peak apex of rounded peak pairs), interval_tf = NULL (minimum and maximum tailing factor), min_fwhm = NULL (minimum full width at half maxima), min_w = 5 (minimum window size of the smoothing function), max_w = 21 (maximum window size of the smoothing function), smooth_method = ‘savgol’ (smoothing method), smooth_times = 2 (number of times the smoother is applied to the data), smooth_win = NULL (set window size of the smoothing function, automatically determined if set to NULL), and max_sigma = NULL (maximum sigma value in the automatic determination of the window size of the smoothing function, otherwise determined from the peak table reported by XCMS).

## 5. Conclusions

The CPC algorithm determines peak quality metrics for peaks detected by the XCMS algorithm. These can be used to filter low quality peaks to reduce their impact on feature correspondence, reduce the number of spuriously matched features, and consequently improve subsequent data analyses and interpretation. We have demonstrated the usability of the approach on authentic data from the guinea pig perilymph metabolome, in which 35% of the peaks detected by XCMS were removed prior to correspondence. These data were obtained from an LC-ESI-QTOF MS instrument. However, the algorithm operates on XCMS objects and is thus also directly applicable to other chromatographic separation and MS techniques. Furthermore, we investigated the ability of the CPC algorithm on mitigating adverse effects of poor XCMS parametrization, often the cause of false peaks, and found consistent improvements when CPC was applied. In a benchmarking experiment, a randomly selected set of 144 peaks was manually curated and the results compared with that of CPC yielding an F1 score of 91.3%. In addition, using multivariate discriminatory data analysis on the same authentic data, we showed that the CPC peak filtering does not negatively impact the biological inference that can be drawn from the data. Finally, the reported peak characteristics can also be used to prioritize detected features of interest for identification. The algorithm is fully integrated into the XCMS workflow and is made available as an R-package (installation instructions are available at https://www.github.com/krispir/cpc/, accessed on 28 January 2022).

## Figures and Tables

**Table 1:** Overview of the results from processing the guinea pig dataset with and without CPC peak filtering.

Dataset	No. Detected Peaks in All Injections	% Filled Peaks in the Dataset	% of Peaks Associated to a Feature	No. Features	% of Features with RSD_QC_ ≤ 30%
Without CPC filtering	130,351	13.7%	49.3%	1270	85.7%
With CPC filtering	84,936	18.9%	69.1%	1213	87.0%

## Data Availability

The raw data used in the example application of this manuscript is available via the MetaboLights repository (https://www.ebi.ac.uk/metabolights/MTBLS2418/, accessed on 28 January 2022).

## Supplementary Materials

The following are available online at https://www.mdpi.com/article/10.3390/metabo12020137/s1, Supplementary text with experimental conditions for the sample preparation of guinea pig perilymph and LC-ESI-Q-TOF/MS based sample analysis. Figures S1–S4: Peaks kept by the CPC algorithm selected from lowest (Figure S1), second lowest (Figure S2), second highest (Figure S3), and highest (Figure S4) quartile of the signal-to-noise ratio, Figures S5–S12: Peaks removed by the CPC algorithm due to not being detected (Figure S5), having a low signal-to-noise ratio (Figure S6), being too narrow (Figure S7), having too low an intensity (Figure S8), having too low a signal-to-noise ratio in combination with being too narrow (Figure S9), having too low a signal-to-noise ratio in combination with having too low an intensity (Figure S10), being too narrow in combination with having too low an intensity (Figure S11), and having a too low signal-to-noise ratio, being too narrow, and having too low an intensity (Figure S12). Figures S13–S15: Results from varying the bw setting during correspondence showing the number of peaks associated with a feature with and without CPC filtering (Figure S13), the number of filled peaks with and without CPC filtering (Figure S14), and the number of features associated with more peaks than there are samples (>15, Figure S15). Figures S16–S19: Panels of 144 randomly selected peaks (36 peaks from each quartile of XCMS reported peak intensities) for benchmarking by manual curation selected from the lowest quartile (Q1, Figure S16), second lowest quartile (Q2, Figure S17), second highest quartile (Q3, Figure S18), and the highest quartile (Q4, Figure S19). Table S1: Outcome of features associated with peaks removed by CPC. Table S2: Benchmarking results from a random selection of 144 peaks subjected to manual curation.
