# Supplementary material for: Comprehensive Peak Characterization (CPC) in Untargeted LC–MS Analysis
Source: Metabolites. 2022 Feb 2;12(2):137. doi: 10.3390/metabo12020137 (PMC8878835; doi:10.3390/metabo12020137)
Supplement: Supplementary file 1 [file metabolites-12-00137-s001.zip › metabolites-1522005-supplementary.pdf]

Supplementary material

Comprehensive Peak Characterization (CPC) in untargeted LC-MS analysis

Kristian Pirttilä [1,*], David Balgoma [1], Johannes Rainer [2], Curt Pettersson [1], Mikael Hedeland [1] and Carl Brunius [3,4]

[1] Department of Medicinal Chemistry, Uppsala University, SE-75123 Uppsala, Sweden; david.balgoma@ilk.uu.se (D.B.); curt.pettersson@ilk.uu.se (C.P.); mikael.hedeland@ilk.uu.se (M.H.)
[2] Institute for Biomedicine, Eurac Research, Affiliated Institute of the University of Lübeck, 39100 Bolzano, Italy; johannes.rainer@eurac.edu
[3] Department of Biology and Biological Engineering, Chalmers University of Technology, SE-41296 Gothenburg, Sweden; carl.brunius@chalmers.se
[4] Chalmers Mass Spectrometry Infrastructure, Chalmers University of Technology, SE-41296 Gothen-burg, Sweden

[*] Correspondence: kristian.pirttila@ilk.uu.se

Supplementary text

Sample preparation of guinea pig perilymph

The sample preparation protocol is presented in detail in a previous publication [1]. Sample preparation by protein precipitation was performed on ice by addition of 20.0 µL cold acetonitrile (4:1 v/v) to 5.0 µL sample aliquots. The samples were allowed to precipitate in the refrigerator (8 °C) for 30 minutes after which they were centrifuged for 15 minutes (21 000g, 4 °C). The supernatant was analyzed without further treatment.

LC-ESI-Q-TOF/MS sample analysis

The sample analysis protocol is presented in detail in a previous publication [1]. Chromatographic separation was performed using a Waters BEH Amide (50x2.1 mm i.d., 1.7 µm particle size, 100 Å pore size) column fitted with a Waters VanGuard BEH Amide pre-column (5x2.1 mm i.d., 1.7 µm particle size, 100 Å pore size). Mobile phases were (A) 95:5 acetonitrile:water (v/v) with 10 mM ammonium formate buffer (adjusted to pH 3 using formic acid) and (B) 5:95 acetonitrile:water (v/v) with 10 mM ammonium formate buffer (adjusted to pH 3 using formic acid). Gradient elution was achieved using a gradient from 0%B to 61%B over 14 minutes using a non-linear concave gradient followed by a 2.7 minute wash out period at 61%B, a fast gradient back to 0%B over 3 minutes, and equilibration at 0%B for 6 minutes. The column temperature was kept at 40 °C and the injection volume was 4.0 µL.

References

1. Pirttilä, K.; Videhult Pierre, P.; Haglöf, J.; Engskog, M.; Hedeland, M.; Laurell, G.; Arvidsson, T.; Pettersson, C. An LCMS-Based Untargeted Metabolomics Protocol for Cochlear Perilymph: Highlighting Metabolic Effects of Hydrogen Gas on the Inner Ear of Noise Exposed Guinea Pigs. *Metabolomics* **2019**, *15*, 138, doi:10.1007/s11306-019-1595-1.

Supplementary figures

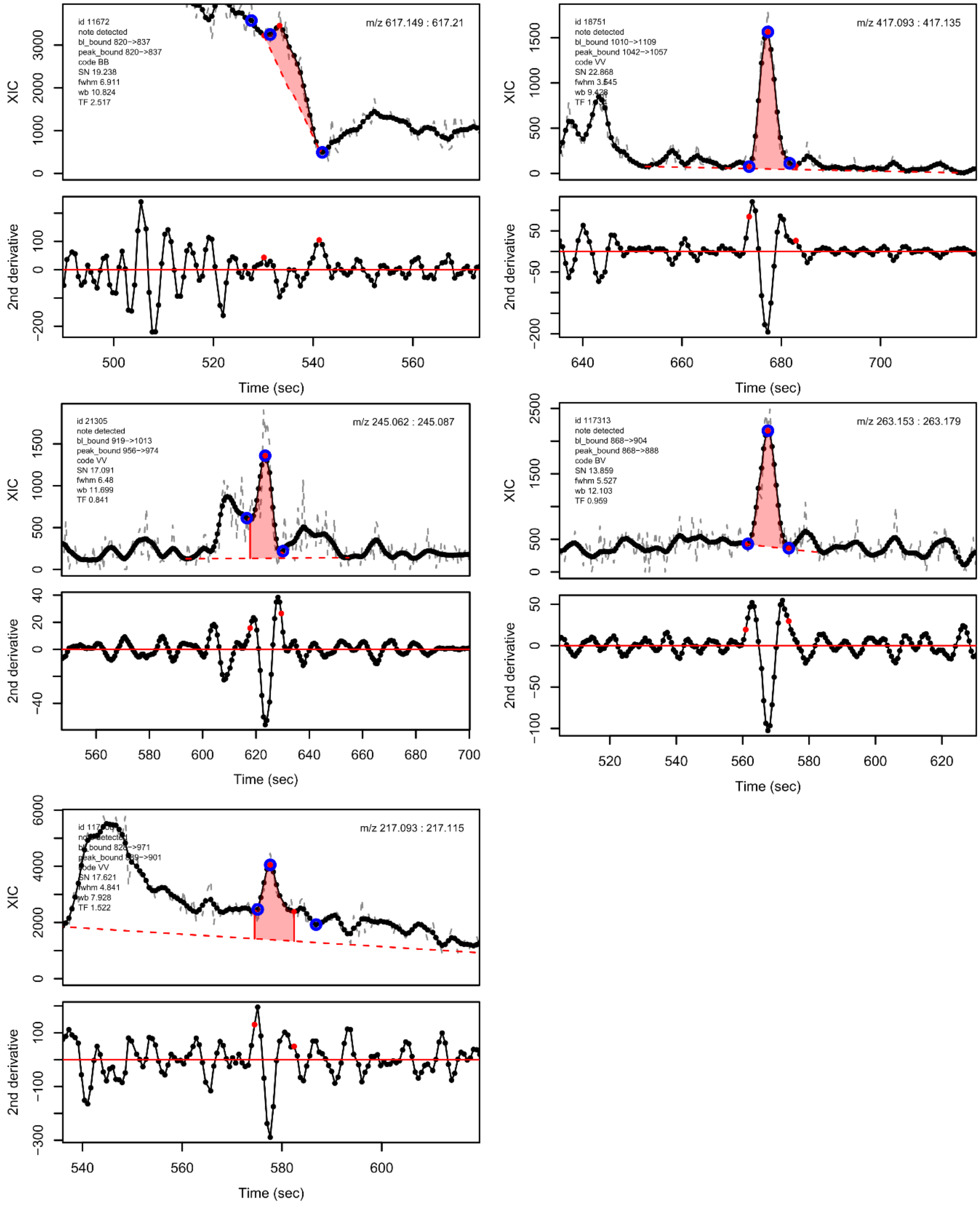

Figure S1 Randomly selected peaks that were kept by the CPC algorithm. These peaks were selected from the lowest quartile of signal-to-noise ratios (S/N < 26.13).

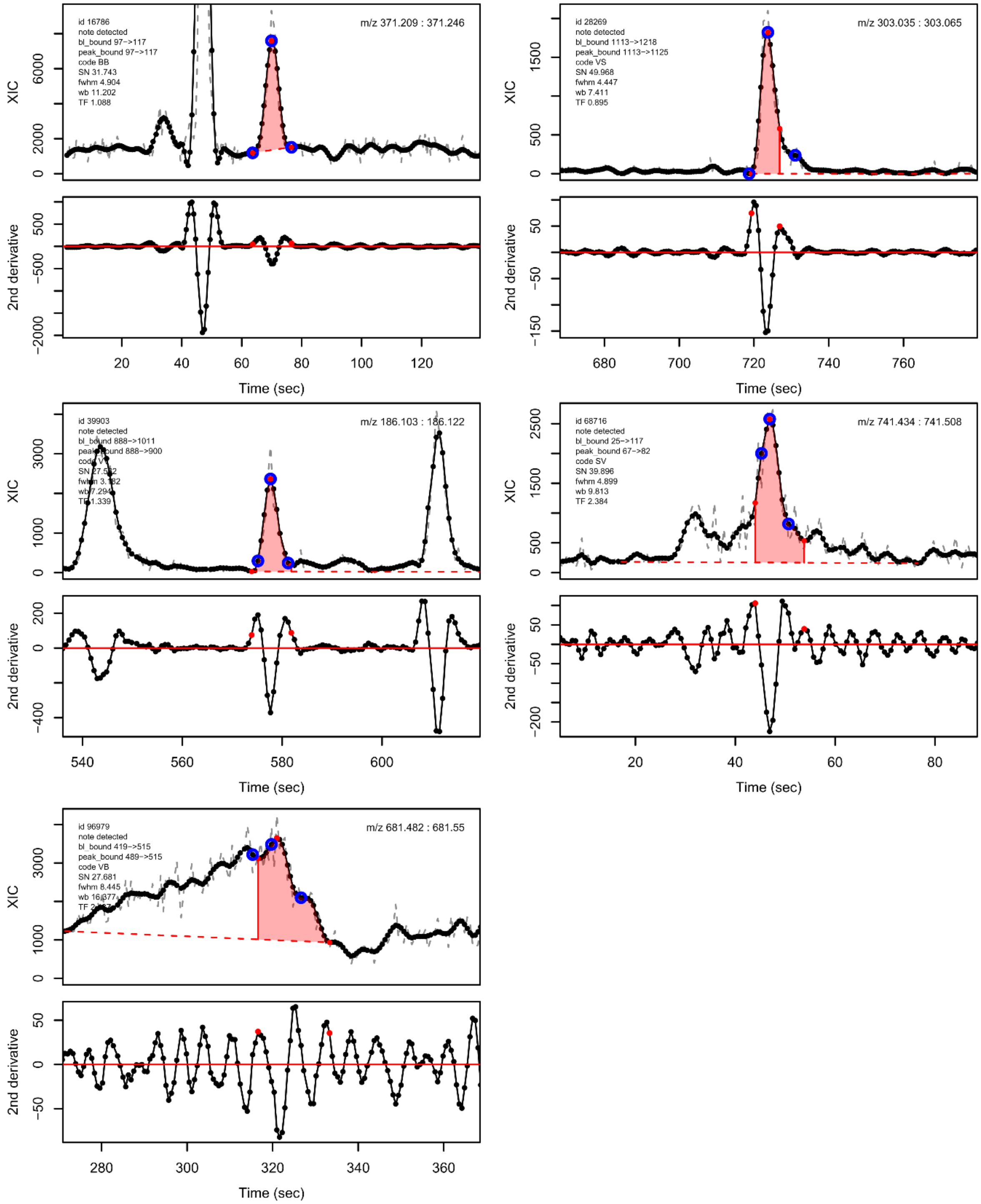

Figure S2 Randomly selected peaks that were kept by the CPC algorithm. These peaks were selected from the second lowest quartile of signal-to-noise ratios (26.13≤ S/N <52.12).

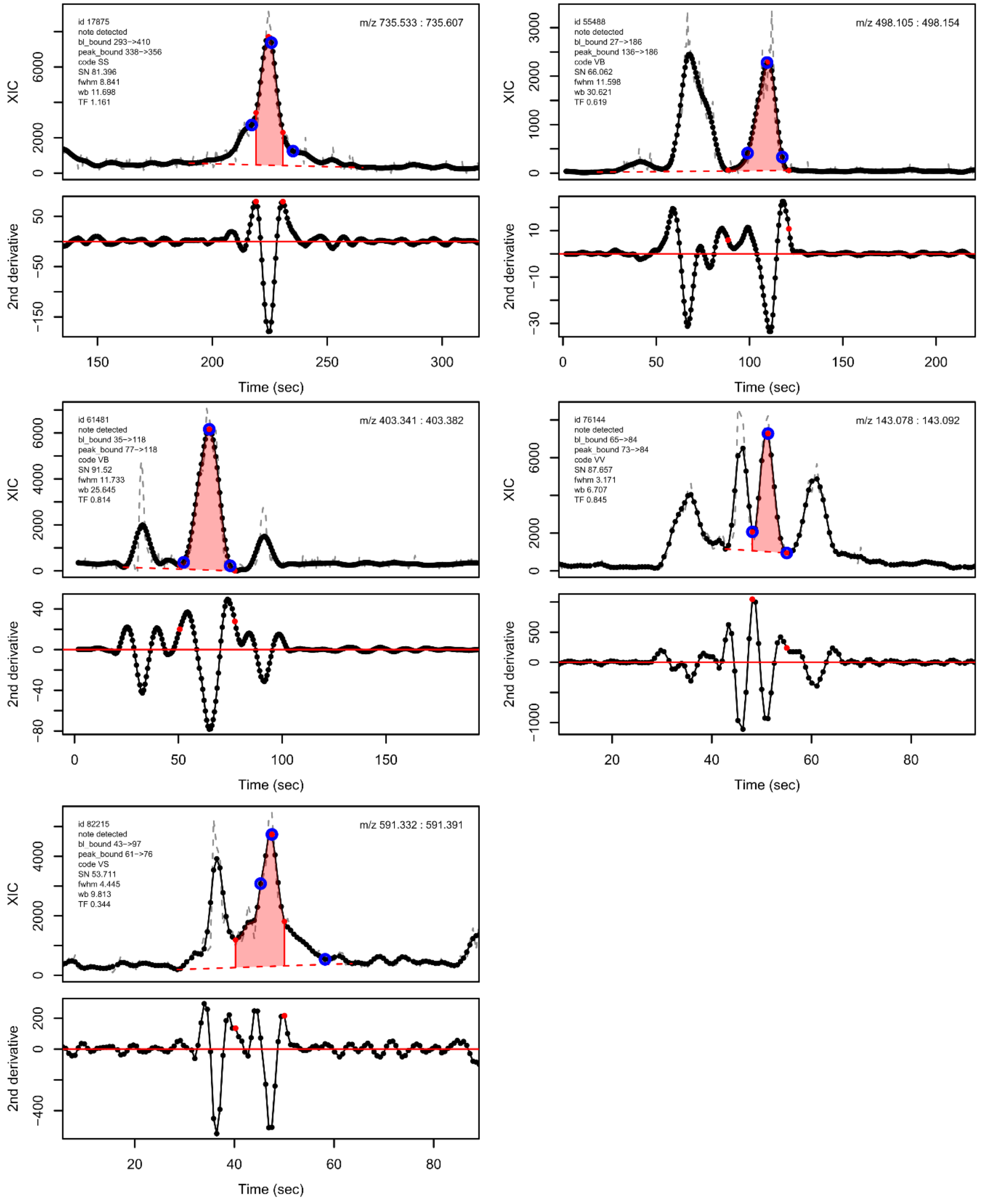

Figure S3 Randomly selected peaks that were kept by the CPC algorithm. These peaks were selected from the second highest quartile of signal-to-noise ratios (52.12 ≤ S/N < 117.4).

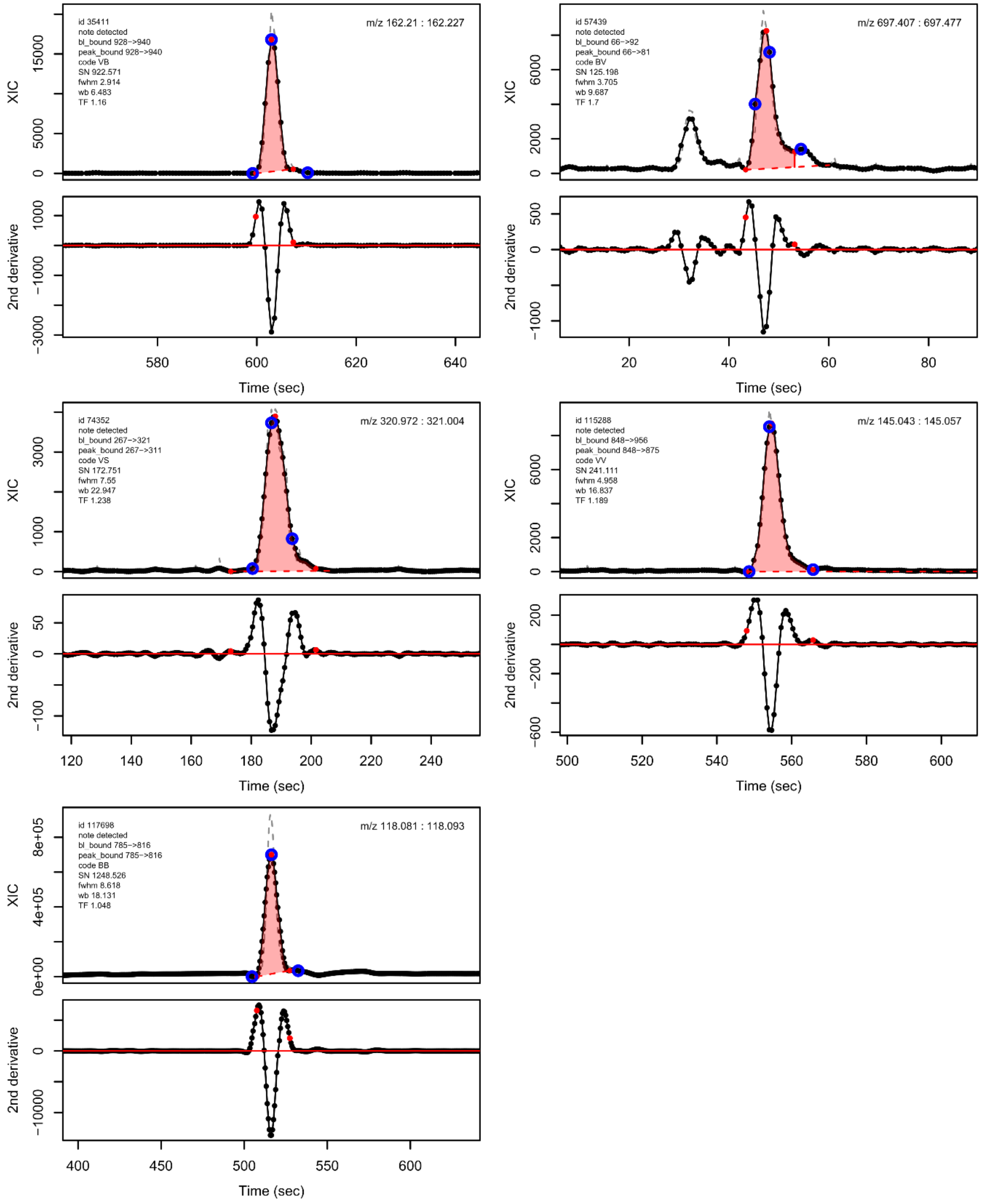

Figure S4 Randomly selected peaks that were kept by the CPC algorithm. These peaks were selected from the highest quartile of signal-to-noise ratios (117.4 ≤ S/N).

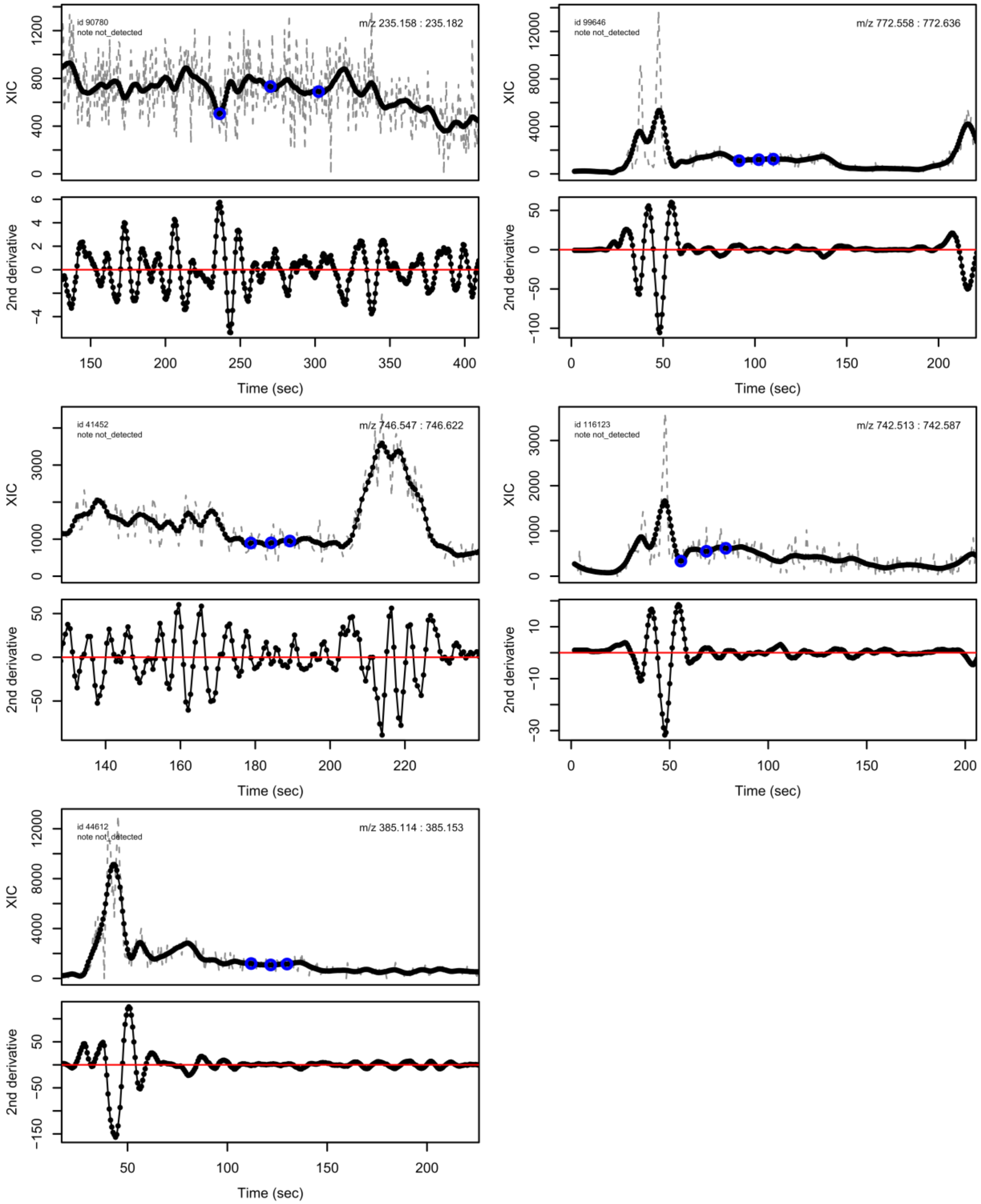

Figure S5 Randomly selected examples of peaks removed by the CPC algorithm because the characteristic peak pattern could not be detected in the second derivative. These were reported by CPC as not detected.

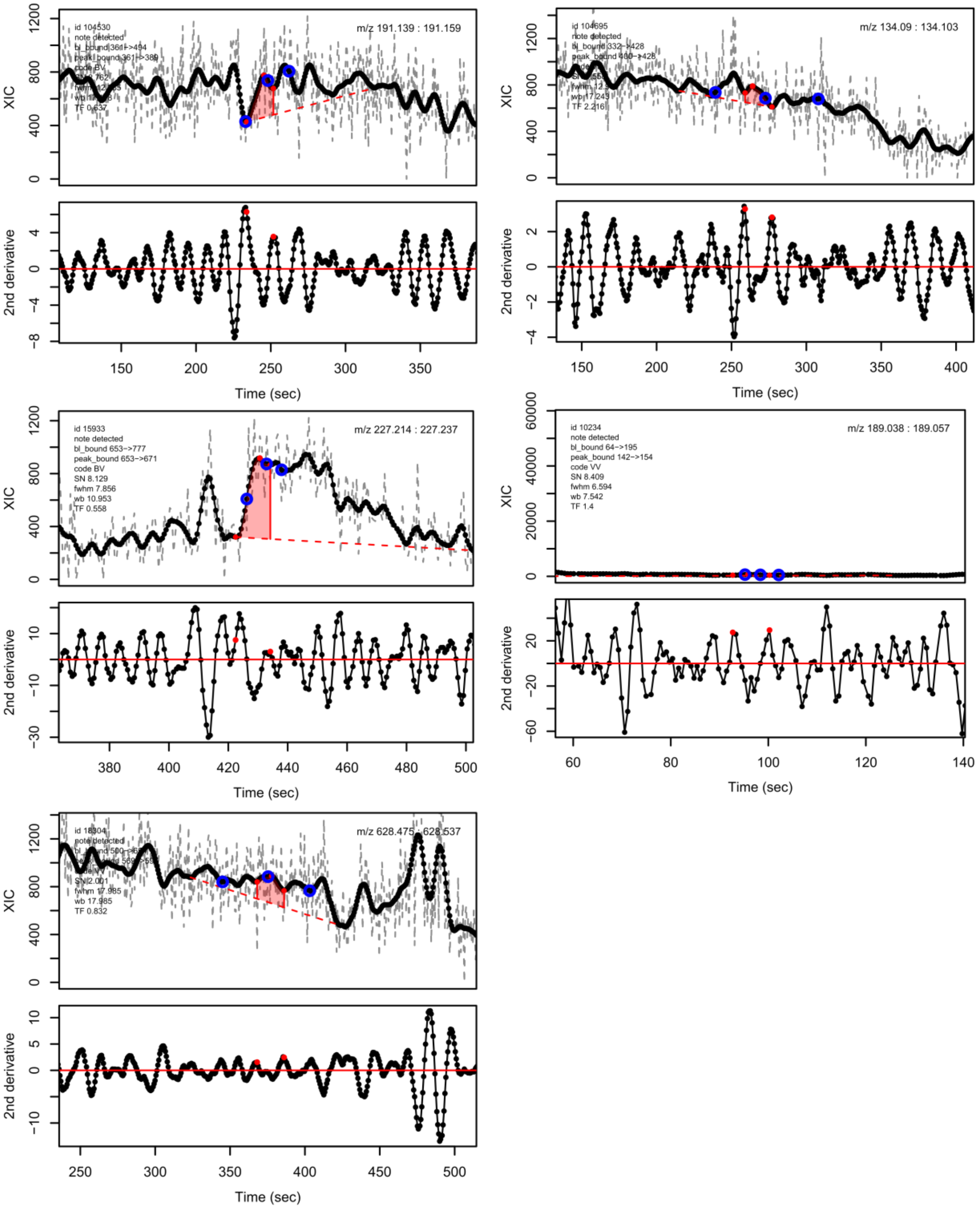

Figure S6 Randomly selected examples of peaks removed by the CPC algorithm due to low signal-to-noise.

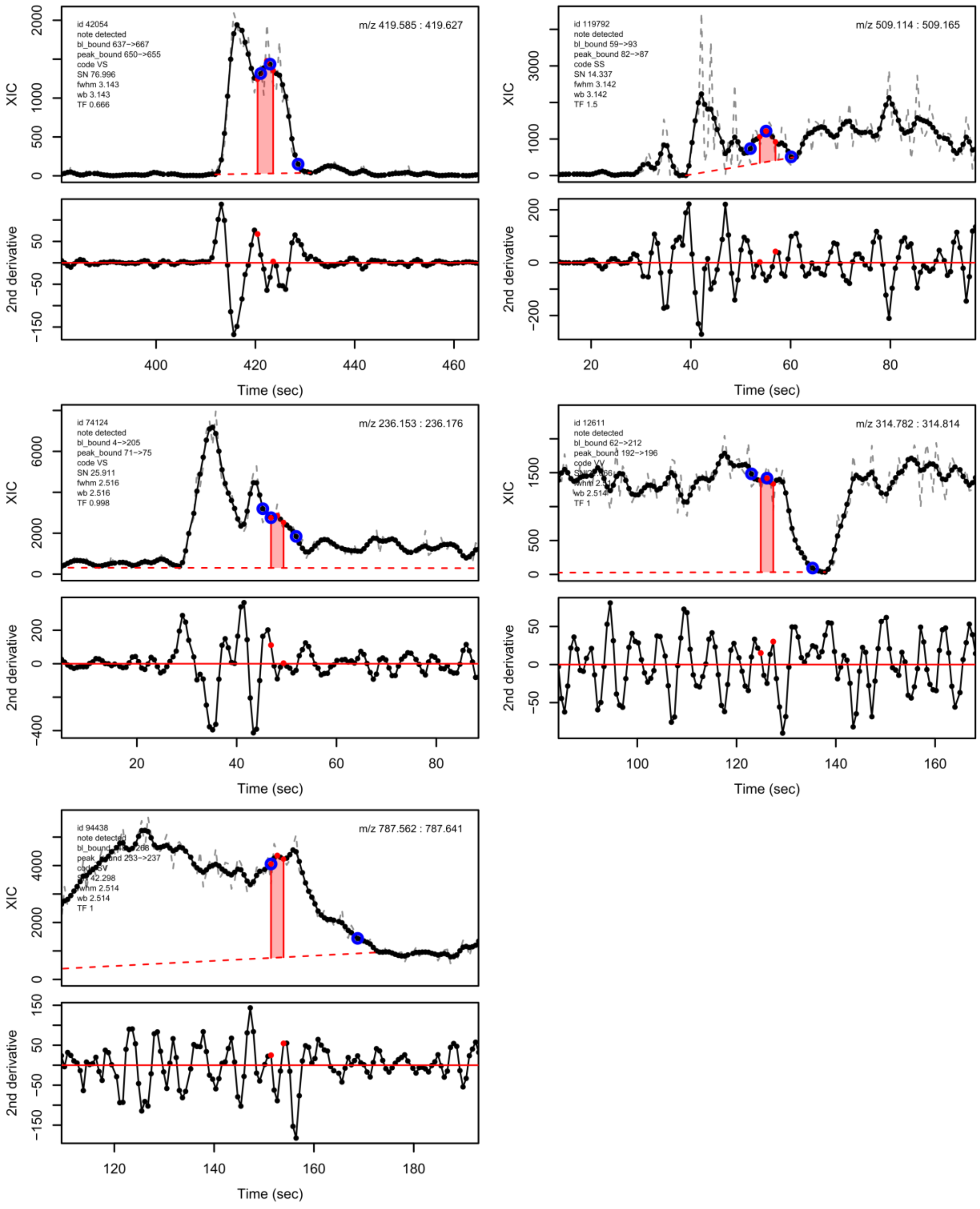

Figure S7 Randomly selected examples of peaks removed by the CPC algorithm due to being too narrow (too few points along the peak).

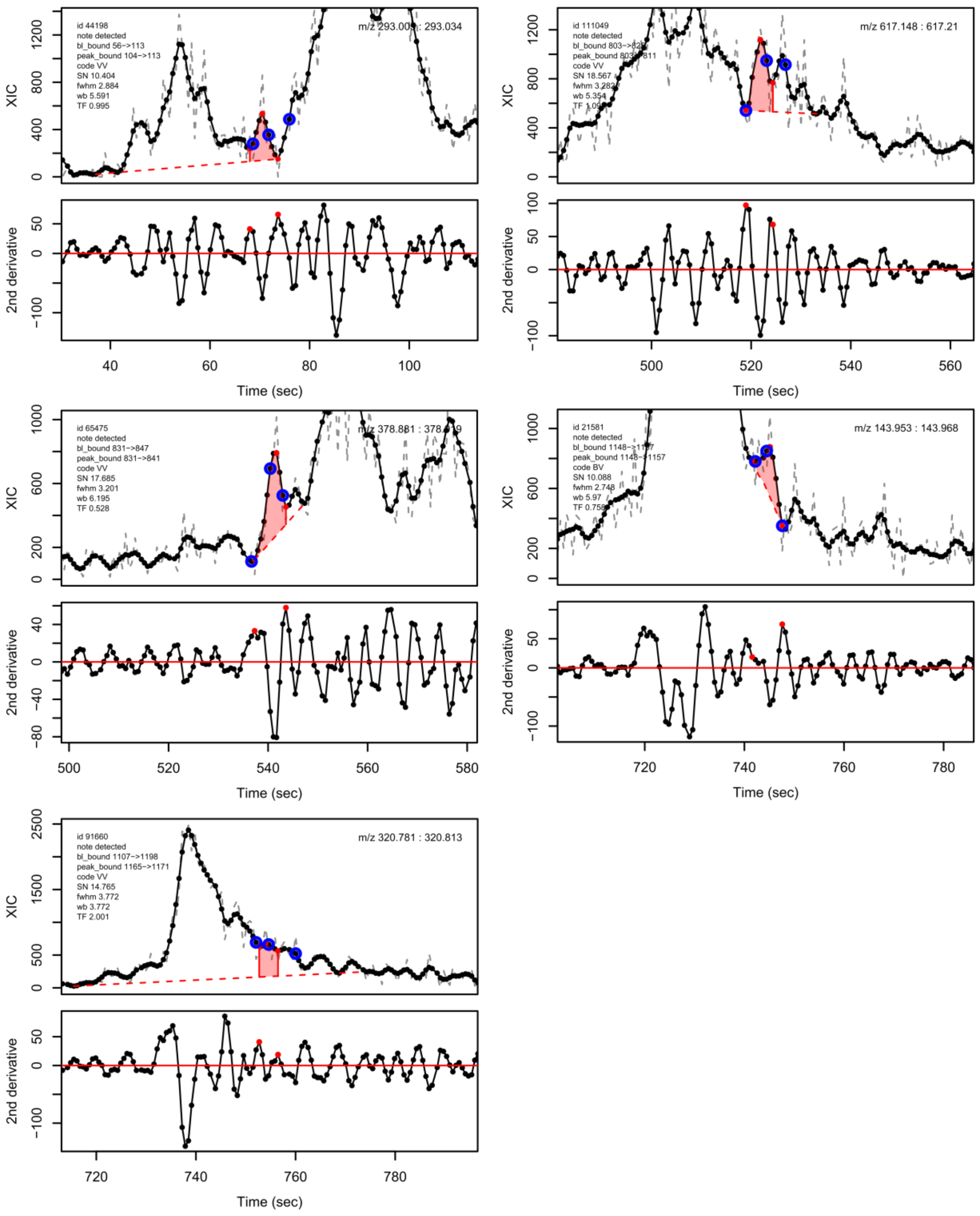

Figure S8 Randomly selected examples of peaks removed by the CPC algorithm due to low intensity.

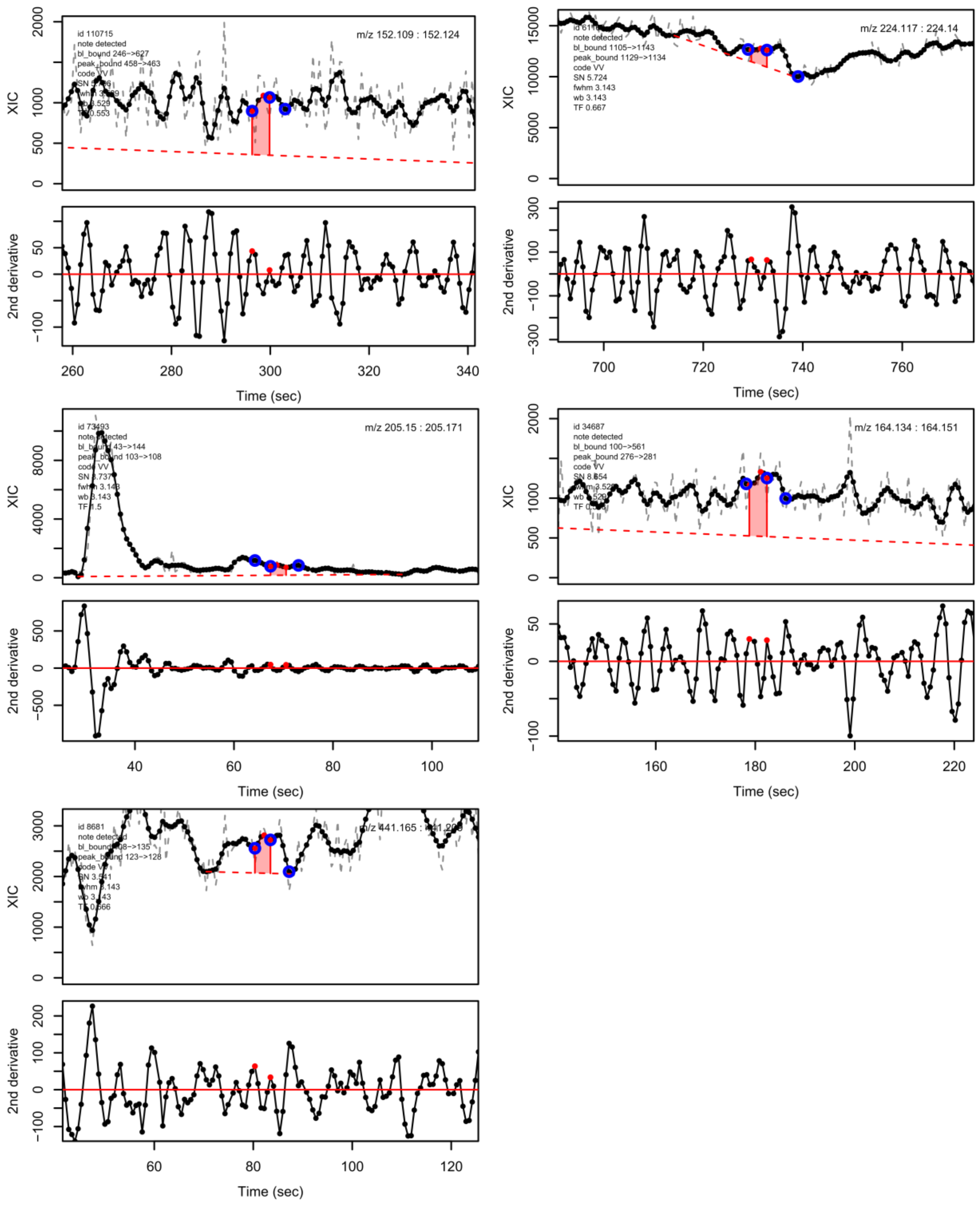

Figure S9 Randomly selected examples of peaks removed by the CPC algorithm due to a combination of low signal-to-noise and being too narrow (too few points along the peak).

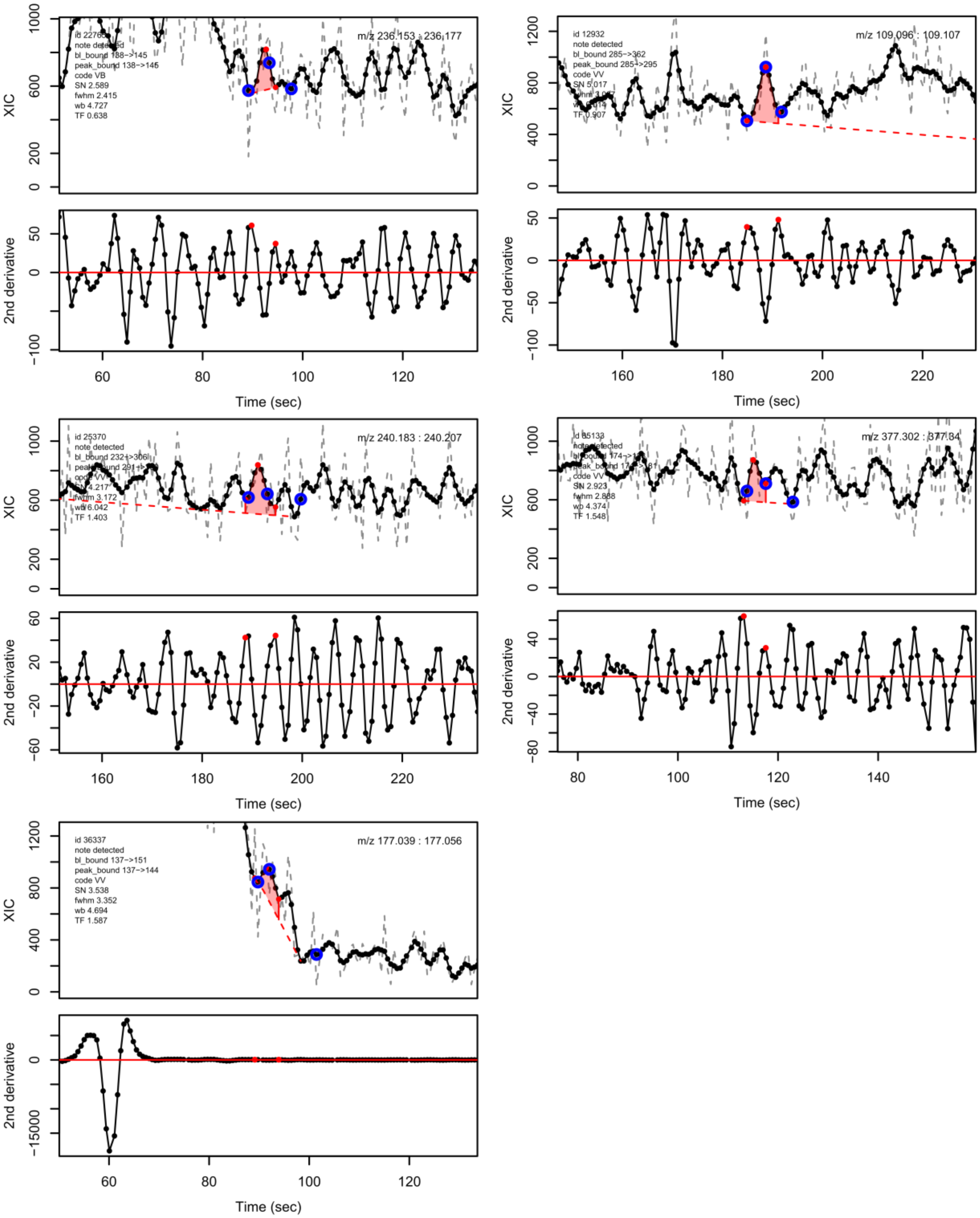

Figure S10 Randomly selected examples of peaks removed by the CPC algorithm due to a combination of low signal-to-noise and low intensity.

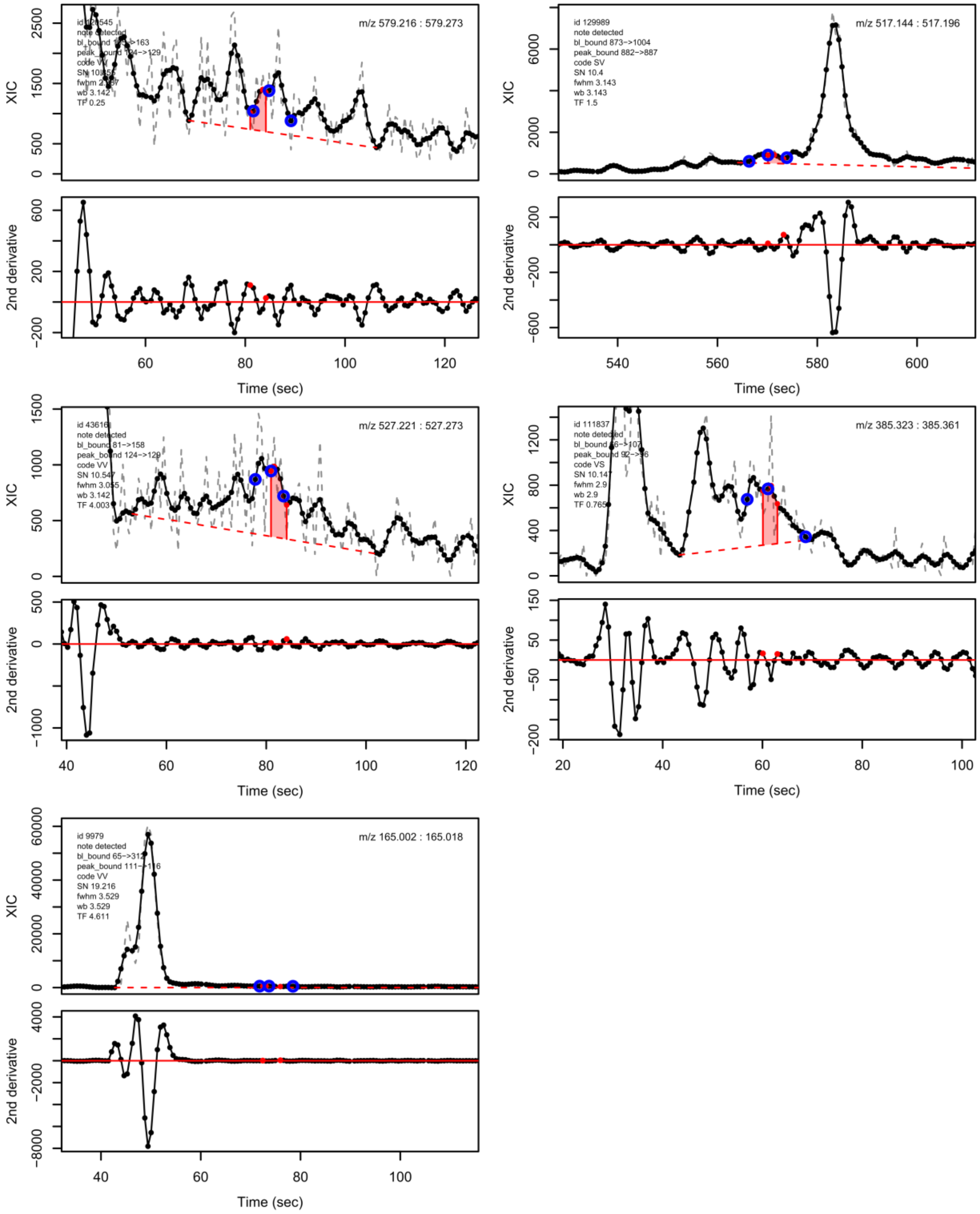

Figure S11 Randomly selected examples of peaks removed by the CPC algorithm due to a combination of them being too narrow (too few points along the peak) and low intensity.

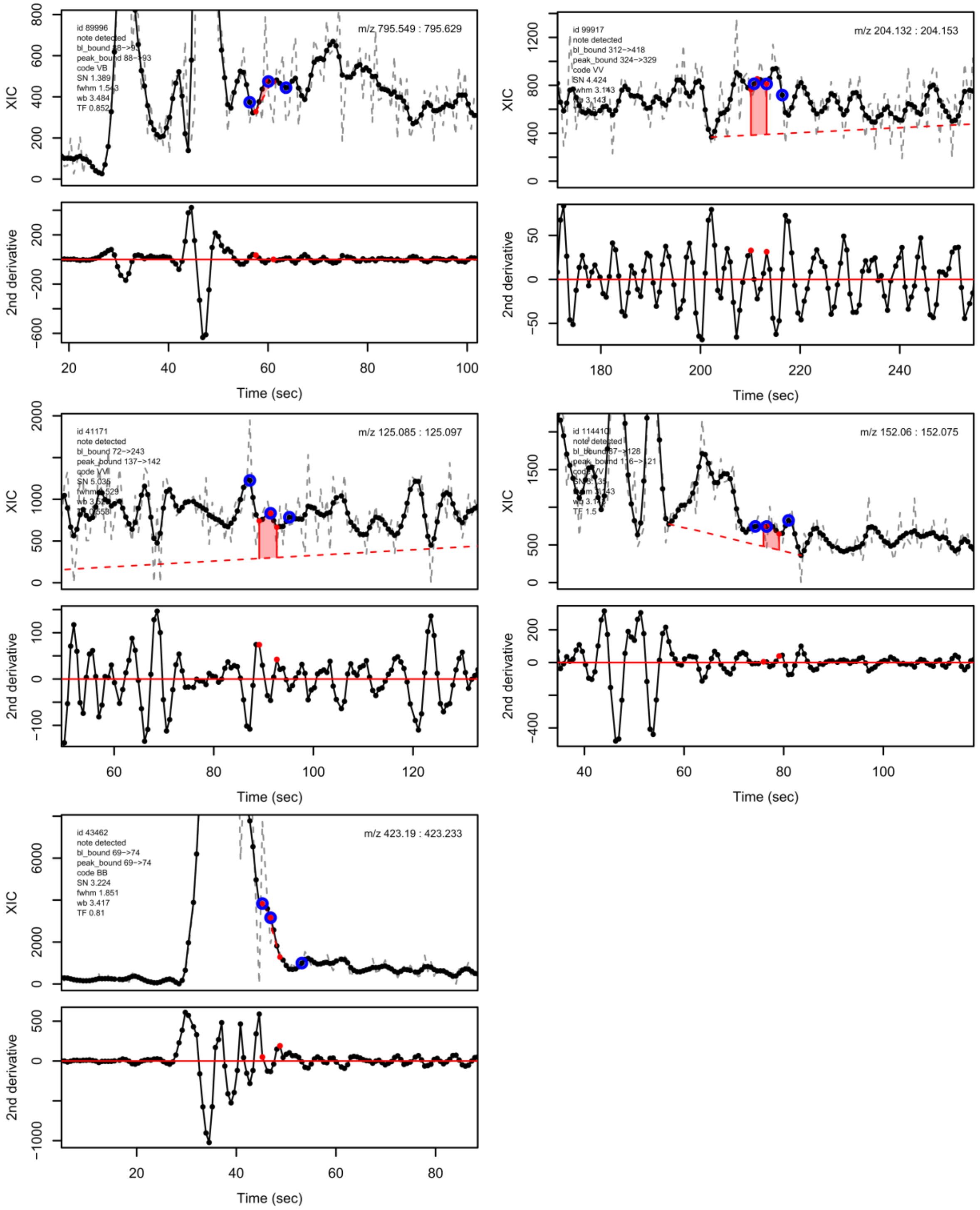

Figure S12 Randomly selected examples of peaks removed by the CPC algorithm due to a combination of low signal-to-noise, being too narrow (too few points along the peak) and low intensity.

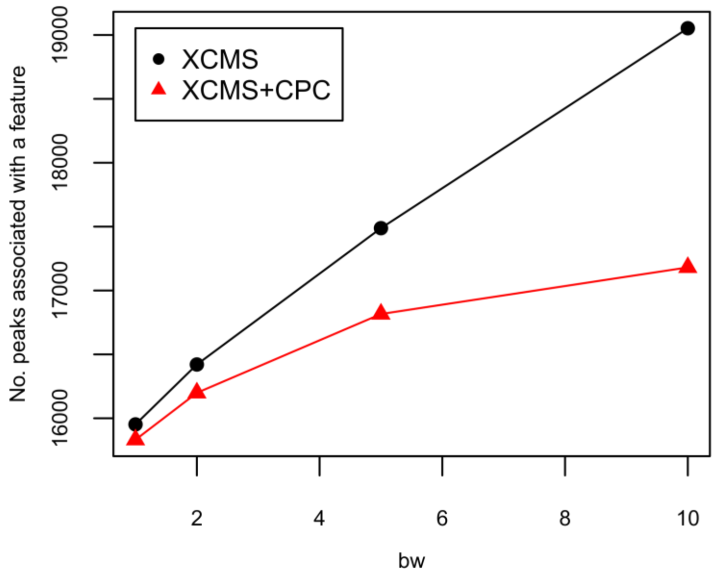

Figure S13 Number of detected peaks that are associated with a feature after correspondence analysis with varying bw parameter setting for data processed using only XCMS (black circles) and a combination of XCMS and CPC (red triangles).

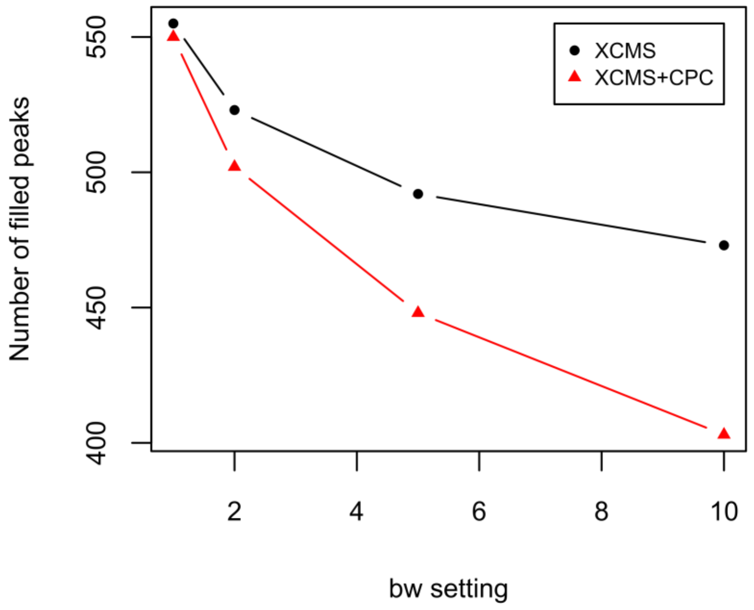

Figure S14 Number of filled peaks with varying bw parameter setting during correspondence for data processed using only XCMS (black circles) and a combination of XCMS and CPC (red triangles).

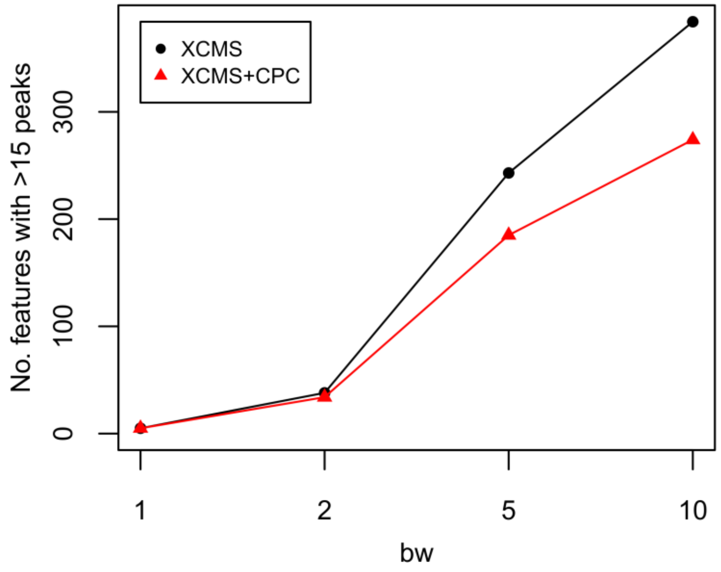

Figure S15 Number of features with more than 15 peaks associated with them with varying bw parameter setting in the correspondence from data processed with only XCMS (black circles) and a combination of XCMS and CPC (red triangles).

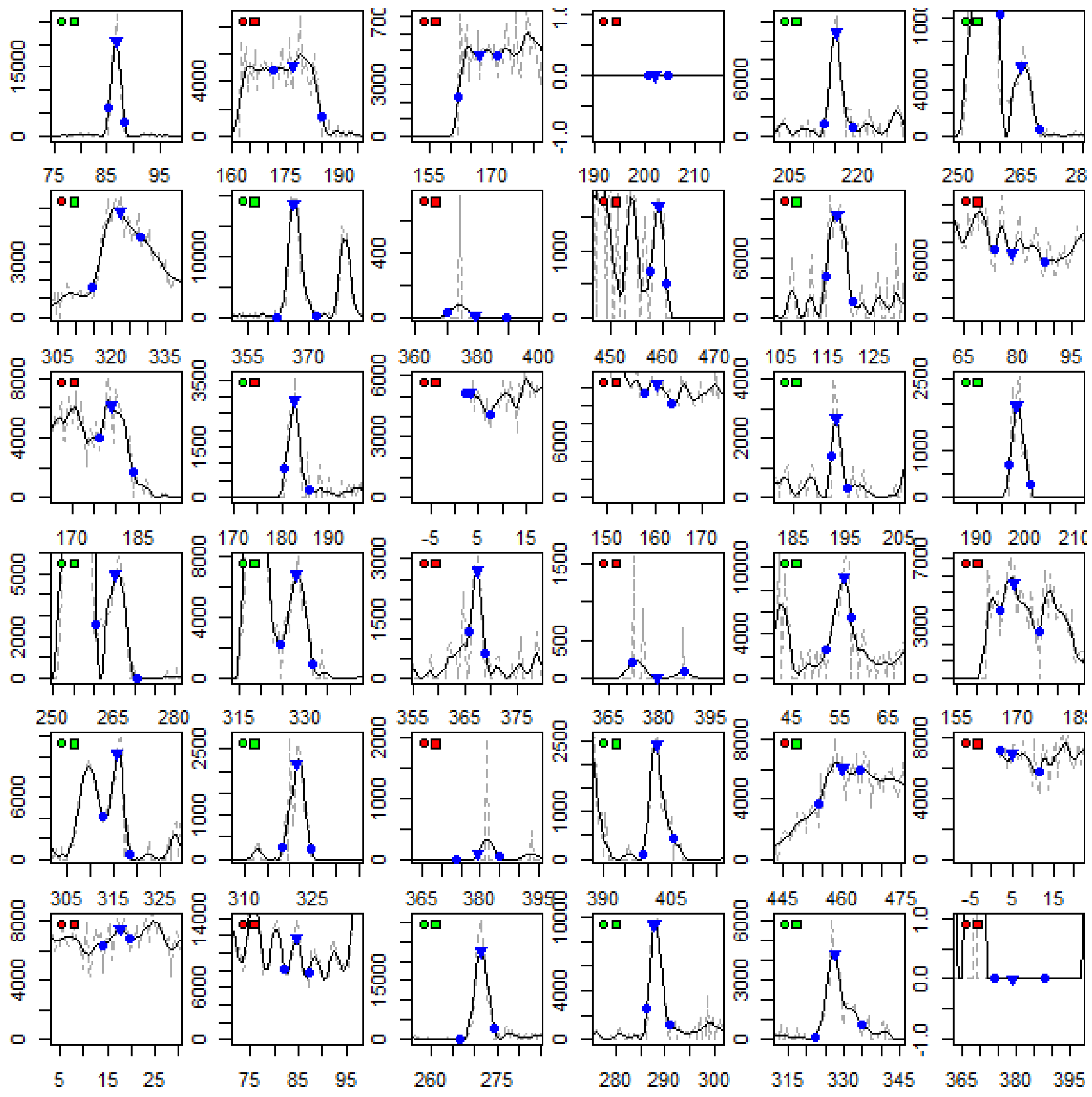

Figure S16 Peaks selected from the lowest quartile of XCMS reported peak intensities. The indicators (top-left corner of each plot) shows the manual classification (filled circles) and the CPC filtering outcome (filled square). A green indicators denotes a peak that should be kept and a red indicator denotes a peak that should be removed and/or was removed by CPC.

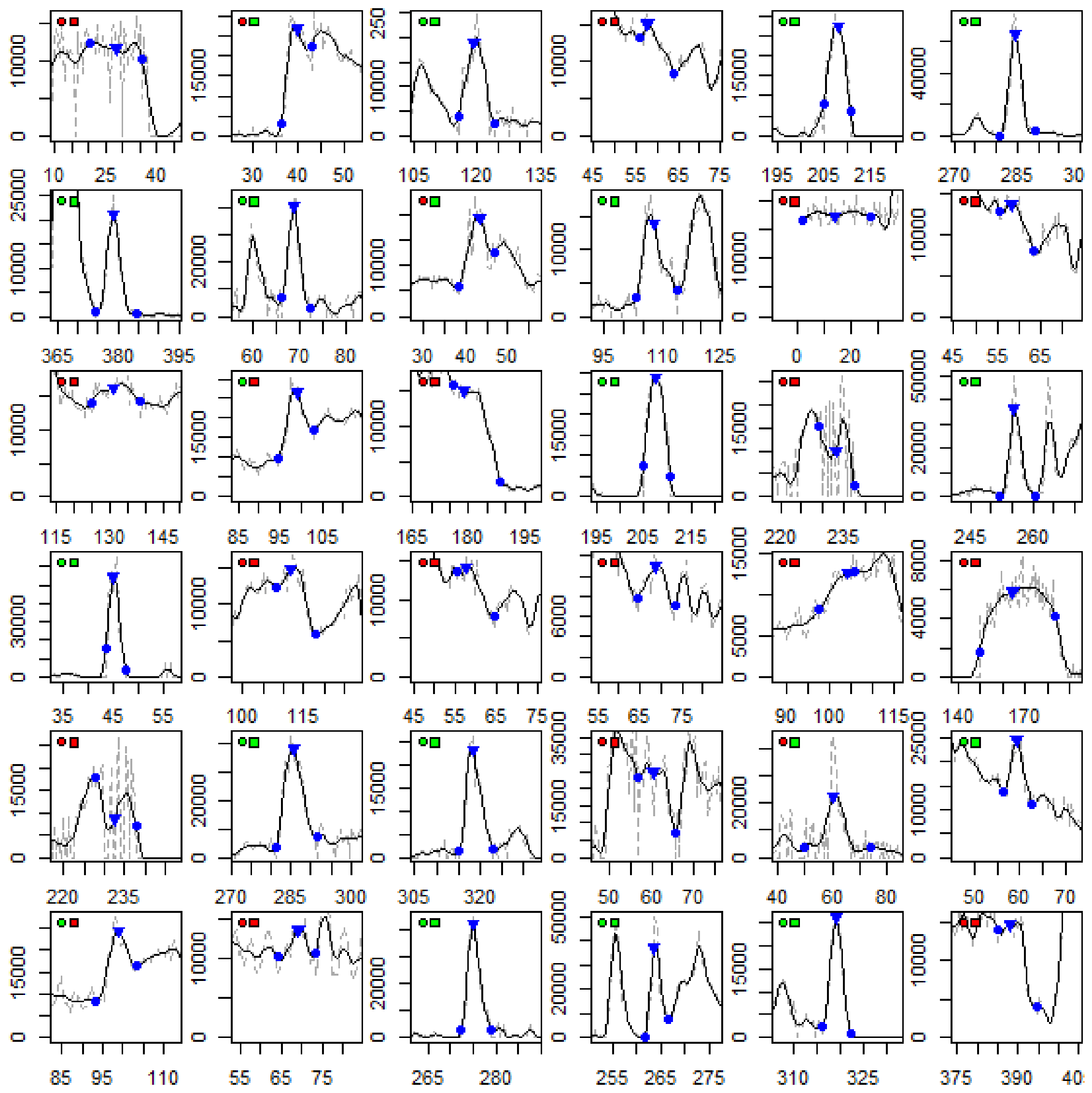

Figure S17 Peaks selected from the second lowest quartile of XCMS reported peak intensities. The indicators (top-left corner of each plot) shows the manual classification (filled circles) and the CPC filtering outcome (filled square). A green indicators denotes a peak that should be kept and a red indicator denotes a peak that should be removed and/or was removed by CPC.

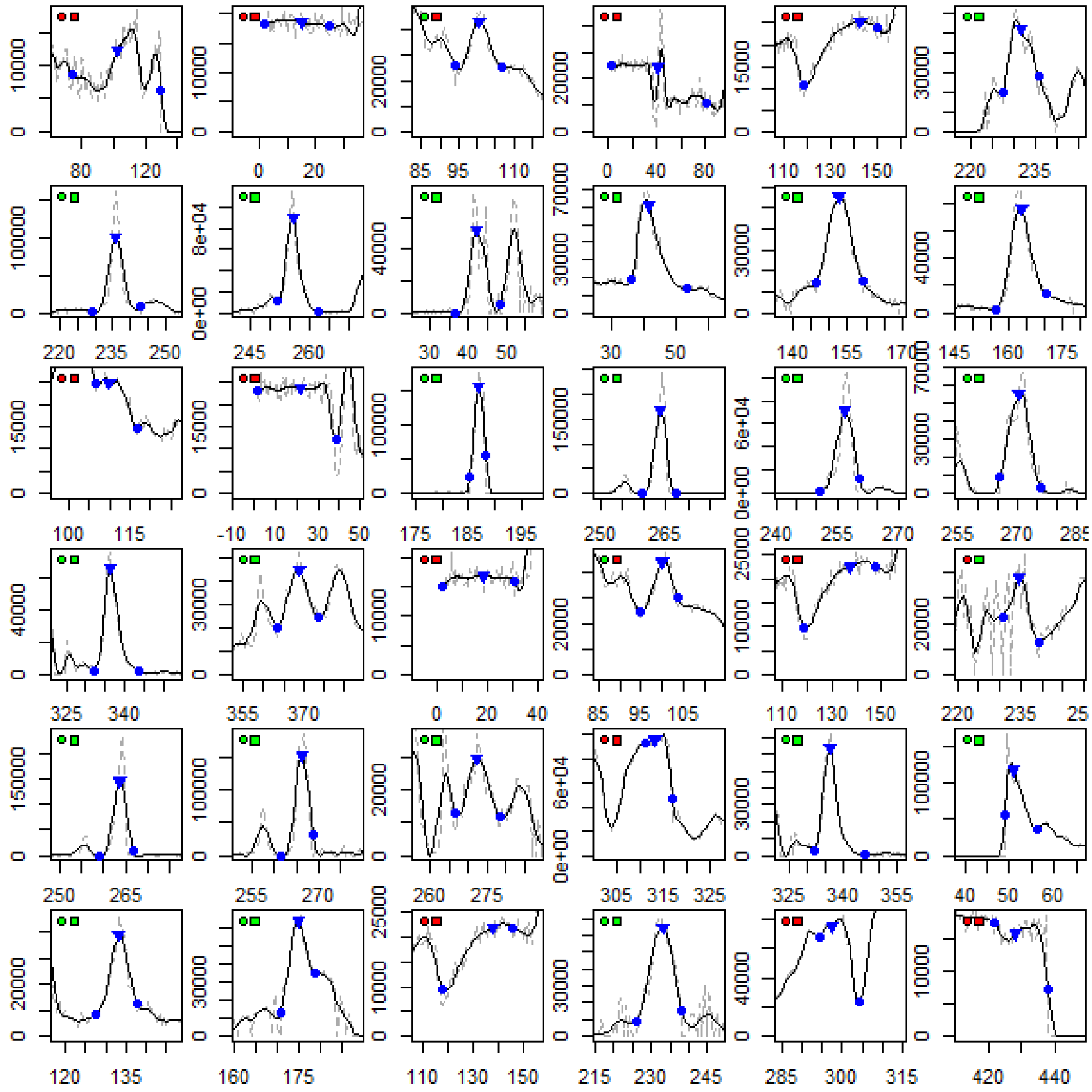

Figure S18 Peaks selected from the second highest quartile of XCMS reported peak intensities. The indicators (top-left corner of each plot) shows the manual classification (filled circles) and the CPC filtering outcome (filled square). A green indicators denotes a peak that should be kept and a red indicator denotes a peak that should be removed and/or was removed by CPC.

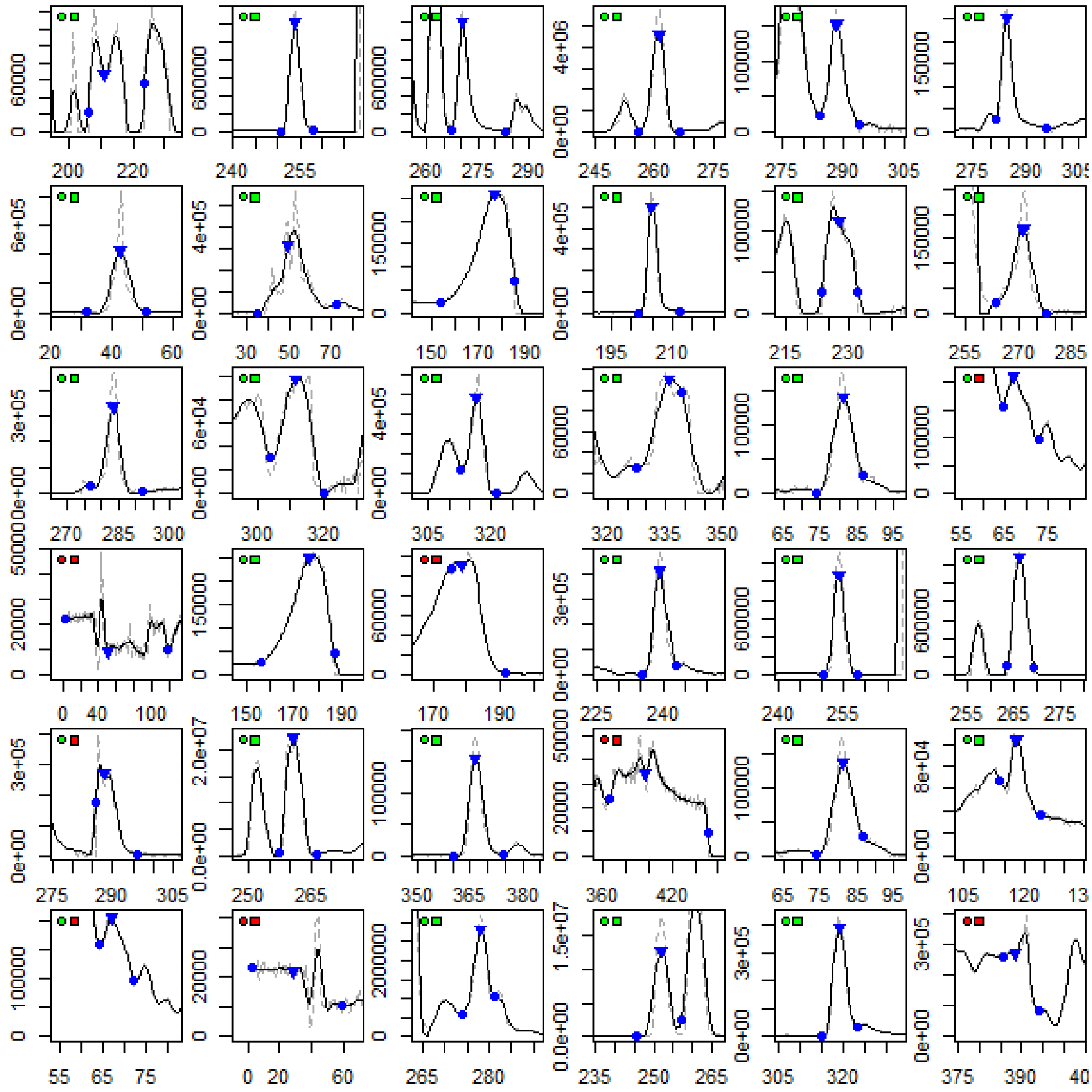

Figure S19 Peaks selected from the highest quartile of XCMS reported peak intensities. The indicators (top-left corner of each plot) shows the manual classification (filled circles) and the CPC filtering outcome (filled square). A green indicators denotes a peak that should be kept and a red indicator denotes a peak that should be removed and/or was removed by CPC.

Supplementary tables

Table S1 The outcome of features that are affected by the CPC filtering. As peaks, that would otherwise have been associated with a feature, are removed by CPC, the affected features may either survive or be removed. If all the peaks that were not removed by CPC are still all associated with the same feature, this is classified as a perfect match. If more than 75% of the peaks that were not removed by CPC are still associated with the same feature, this is classified as a partial match. With less matching peaks than this, the feature is considered removed. Results derived from processing all QC sample injections (n = 15).

	**bw parameter setting**			
**Outcome of features affected by CPC peak filtering**	**1**	**2**	**5**	**10**
All retained peaks still associated with the same feature, remaining peaks from XCMS peak filling	11	12	92	171
All retained peaks still associated with the same feature, other detected peaks make up the rest			2	9
All retained peaks still associated with the same feature, remaining peaks from XCMS peak filling AND other detected peaks			1	1
≥75% of retained peaks still associated with the same feature, remaining peaks from XCMS peak filling			1	6
≥75% of retained peaks still associated with the same feature, other detected peaks make up the rest				1
≥75% of retained peaks still associated with the same feature, remaining peaks from XCMS peak filling AND other detected peaks				4
All retained peaks are distributed into other features		1	6	11
Some of the retained peaks are distributed into other features				1
None of the retained peaks are associated with a feature	8	13	22	33

Table S2 Benchmarking results based on a random selection of 36 peaks from each quartile of XCMS reported peak intensities for a total of 144 peaks. Each peak was subjected to an expert assessment by the first author based on the apparent shape of the peak, the approximate signal-to-noise ratio, and the width of the peak followed by classification as a true or false peak. The result from the manual classification was then contrasted with that of the CPC processing. Panels of all the inspected peaks are presented in Figures S16-S19.

**Metric**	**Formula**	**Q1**	**Q2**	**Q3**	**Q4**	**Overall**
No. manually filtered peaks		20	19	13	5	57
No. manually kept peaks		16	17	23	31	87
True Positive (TP)		15	15	21	28	79
True Negative (TN)		17	16	12	5	50
False Positive (FP)		3	3	1	0	7
False Negative (FN)		1	2	2	3	8
True Positive Rate (TPR)	$TPR = \frac{TP}{TP + FN}$	93.8%	88.2%	91.3%	90.3%	90.8%
True Negative Rate (TNR)	$TNR = \frac{TN}{FP + TN}$	85.0%	84.2%	92.3%	100%	87.7%
Accuracy (ACC)	$ACC = \frac{TP + TN}{TP + FP + TN + FN}$	88.9%	86.1%	91.7%	91.7%	89.6%
F1 score	$F_1 = \frac{2TP}{(2TP) + FP + FN}$	88.2%	85.7%	93.3%	94.9%	91.3%
